# Determinants of teenage pregnancy in Degua Tembien District, Tigray, Northern Ethiopia: A community-based case-control study

**DOI:** 10.1371/journal.pone.0200898

**Published:** 2018-07-25

**Authors:** Brhane G/kidan Ayele, Tesfay Gebrehiwot Gebregzabher, Tesfay Tekle Hailu, Belete Abera Assefa

**Affiliations:** 1 Public Health Department, Public Health Emergency Management Directorate, Tigray Health Research Institute, Mekelle, Ethiopia; 2 School of Public Health, Mekelle University, Mekelle, Ethiopia; TNO, NETHERLANDS

## Abstract

**Background:**

Approximately 16 million teenagers aged 15–19 years and 2 million teenagers under the age of 15 years give birth annually, with 95% of these births occurring in developing countries. Ethiopia has one of the highest teenage fertility rates in Sub-Saharan Africa; however determinants of teenage pregnancy are not well studied. Therefore, this study aimed to identify determinants of teenage pregnancy among female teenagers in Degua Tembien district, Tigray, Northern Ethiopia, in 2015.

**Methods:**

A community-based case-control study was conducted in Degua' Tembien district from February 01, 2015 to March 15, 2015 with a randomly selected total sample size of 414 females (with a ratio of 1:2 case to control, 138 and 276 respectively). Data were entered in to Epi-Info and analyzed using SPSS software. Multivariable logistic regression was used to assess predictors of the outcome variable; variables with a p-value <0.25 in bivariable analysis were included in the model. Statistically significance was considered at a p-value ≤0.05 in both bivariable and multivariable logistic regression analyses.

**Result:**

The mean ages (plus or minus standard deviation (±SD)) of cases and controls were 18.47 (0.72) and 17.09 (1.2) years, respectively. After adjustment for other variables, predictors of teenage pregnancy included: lower monthly income below ~$25 and ~$25–50 (adjusted odds ratio (AOR) = 23.96; 95% confidence interval (95%CI) 4.89–117.29 and AOR = 4.91; 95%CI 1.64–14.66, respectively); aged 18–19 years (AOR = 16.75; 95%CI 6.45–43.47); being married (AOR = 15.91; 95%CI 7.43–34.04); not communicating with parents on reproductive health issues (AOR = 6.52; 95%CI 3.12–13.64) and having a history of maternal teenage pregnancy (AOR = 4.14; 95%CI 1.84–9.33).

**Conclusion:**

The factors associated with teenage pregnancy in our study were lower family monthly income, being married, being in the 18–19 year age group, not communicating with parents on reproductive health issues and having a maternal history of teenage pregnancy. Programs that encourage parent-teenage communication of reproductive health issues, starting from early adolescence, in order to build skills to prevent pregnancy in the late teenage years, are very important. In addition, multi-pronged activities across sectors that encourage delayed marriage and improve health service utilizations for girls are essential.

## Introduction

Approximately 16 million adolescent girls aged 15–19 years and 2 million adolescents under the age of 15 years give birth annually. These births constitute roughly 11% of all births worldwide; nearly 95% occur in developing countries. The proportion of adolescents giving birth ranged from 2% in China, to 18% in Latin America and the Caribbean, to more than 50% in sub-Saharan Africa [1]. Half of all adolescent births occur in just seven countries: Bangladesh, Brazil, the Democratic Republic of Congo, Ethiopia, Nigeria, India and the United States [2]. Each year, births to adolescent girls aged 15 to 19 years account for 16% of all births in sub-Saharan Africa [3].

Giving birth as a teenager leads to having more children than women who start childbearing after their teenage years. The outcome of such phenomenon contributes to high population growth. Teenage pregnancy is also associated with social stigma, stillbirth, low birth weight and maternal death. Furthermore, the complete lack of access to health care, absence of skilled delivery services, or delayed entry into antenatal care (ANC) deprive both the teenage mother and her offspring of basic health care services [4].

In addition, the likelihood of access to education of children born to teenagers is rare. Among those who gain access to education, frequent interruptions and absente may lead to poor academic results; consequently, these children may face unemployment when they grow up and be less physically fit for labor at a later age. Female teenagers’ may be particularly at risk for sexual harassment and rape due to poor physical fitness. Furthermore, their inability to negotiate safer and delayed sexual debut contributes to early pregnancy, as experienced by their mothers. This process continues repeatedly, which contributes to the perpetuation of the vicious cycle of poverty [5–8].

The government of Ethiopia has taken many measures to reduce teenage pregnancy and its consequences. Some of the measures taken thus far include amending and/or implementing: a law against early marriage; a national adolescent and youth reproductive health strategy; legalization of abortion; a youth and HIV/AIDS policy; and community mobilization against harmful traditional practices, including the association of such practices with the new criminal law. However, teenage pregnancy continues to be a burning public health and demographic challenge in Ethiopia [9]. The country has one of the highest adolescent fertility rates in sub-Saharan Africa– 72 births for every 1,000 young women aged 15–19 years [10,11]. Many researches identify different determinant factors of teenage pregnancy: like not living with parents, low socioeconomic status, early sexual intercourse and low level of contraception knowledge [12–14]. Research of determinates of teenage pregnancy in Ethiopia is extremely limited. While at least one study has associated teenage pregnancy with early marriage [11], this single factor alone cannot constitute all determinants of teenage pregnancy. The dearth of information on determinants of teenage pregnancy is particularly pronounced in Degua Tembien district; Tigray regional state supporting the need for further evidence.

Therefore, this study aimed to determine the factors contributing to teenage pregnancy to help policy makers, program managers and health care authorities with better decision making in planning and problem solving.

## Methods

### Study area and period

This study was conducted in Degua' Tembien district from February 01 to March 15, 2015. The estimated total population of the district is 138,334, based on the 2007 census; in 2014/2015, 67,369 (48.7%) were male and 70, 965 (51.3%) female. The district has one newly-upgraded primary hospital, five health centers, 24 health posts, two private drug shops, sixty-one primary schools, one high school and one preparatory school. The primary hospital is the only health facility providing youth-friendly services among all of the health facilities in the district. Degua’ Tembien is one of the least performing districts in terms of family planning and institutional delivery utilization. The contraception acceptance and skilled attendance rates were reported as 47.8% and 35.7%, respectively, which are much lower than the regional averages of 54.8% and 50%, respectively. Moreover, Degua' Tembien is among the districts with the highest stillbirth rate and neonatal death rate, at 2.2% and 0.62%, respectively [15]. Though not documented, from observation, the district has a high rate of teenage pregnancy.

### Study design

A community-based case-control study design was used to assess determinants of teenage pregnancy.

### Source population

Female teenagers in Degua Tembien district constituted the source population.

### Study population

Female teenagers registered in the pre-survey in the study area (290 pregnant and 1608 non-pregnant teenagers) were eligible for participation.

### Study unit

Actual participants included 138 pregnant and 276 non-pregnant teenagers in Degua Tembien district.

### Sample size calculation

$$n1=\frac{{\left(Z1-\alpha /2+Z1-\beta \right)}^{2}2P(1-P)}{{\left(p1-p2\right)}^{2}}\times \frac{c+1}{2c}$$

$$n1=\frac{{\left(Z1-\alpha /2+Z1-\beta \right)}^{2}P(1-P)}{{\left(p1-p2\right)}^{2}}\times \frac{c+1}{c}$$

n2=cxn1

Where:

Z1- α/2 (95%CI) = 1.96

P = Average of P_1_ and P_2_

Z1- β (80% power) = 0.84

P1 = Proportion of exposure among cases P2 = Proportion of exposure among controls C = Ratio of controls to cases = 2:1 n_1_ = Number of cases n_2_ = Number of controls

Based on research conducted in South Africa on risk factors for teenage pregnancy, living with both parents was less common among pregnant teenagers. In that research, a higher proportion (50.7%) of controls was reported to "live with both parents" compared to cases (35.6%) [4]. Sample size was calculated with an assumption of 95%CI, 80% power and a 10% contingency for the non-response rate, using predictor variables such as: forced sexual debut, not living with biological father, being from a non-nuclear family and living with both parents. Of all the variables, the latter—“living with both parents”—was selected as it gives the highest sample size compared to the other variables. Finally, a total sample size of 414, with 138 cases and 276 controls, was utilized in the study (Table 1).

**Table 1:** Sample size calculation for the predictors of teenage pregnancy among female teenagers in Degua Tembien district, Tigray, Northern Ethiopia based on significant factors from other studies.

Significant predictors	Citation	CI	Power	Case:	Proportion of exposure among		OR	Samples size including 10%		
				Control	Cases	Controls		Case	Control	Total
1.Household not a nuclear family	4	95	80	1:02	0.845	0.522	4	38	76	114
2.Not living with biological father	4	95	80	1:02	0.744	0.521	2.62	62	124	186
**3.Biological parents live together**	**4**	**95**	**80**	**1:02**	**0.356**	**0.507**	**0.55**	138*	276*	414*
4.Sexual initiation forced or raped	4	95	80	1:02	0.319	0.181	2.35	123	246	369
5.Primary and below educational level	36	95	80	1:02	0.904	0.791	1.95	136	271	407

*Therefore the total sample size was 414 (cases (n_1_) = 138 and controls (n_2_) = 276).

### Sampling methods and procedures

Cases were pregnant teenagers (between the ages of 13–19 years) at the time of interview and controls were those who had never been pregnant, from the same age group as the cases. Two weeks before the data collection period, a pre-survey was conducted by house-to-house visit to develop a sampling frame for both cases and controls. Finally, cases were selected randomly using a computer-based program (Open-Epi random program) from the already developed sampling frame, after proportional allocation to the health institutions’ catchments (one primary hospital and five health centers), based on the existence of cases. Two controls were also selected randomly for each case from the same "kebelle" (the smallest administrative unit) using the identical random program.

### Data collection techniques and tools

A thorough literature review of risk factors for teenage pregnancy was conducted prior to the development of a questionnaire to identify determinants of teenage pregnancy. Determinants previously identified by other researchers and potential determinants for the local setting was recognized using information on reports from the regional health bureau and district health office. A rough assessment and observation of reproductive health services were conducted. Then a structured interviewer-administered questionnaire on potential determinants of teenage pregnancy was developed in English and translated to the local language of the study area (Tigrigna) prior to the start of the fieldwork. The questionnaire (S1) had three parts. The first part was related to the socio-demographic and socio-economic characteristics of respondents, such as: marital status, living arrangements, family size, family income and educational status of both the study subjects and their parents. The second part was comprised of topics related to sexuality and reproductive health, such as: age at menarche, family history of teenage pregnancy, communication with parents on reproductive health issues, and past and current pregnancy status. In addition to this, questions related to the details of pregnancy, such as: parity, planning of the current pregnancy, and health service utilization (e.g. ANC) were also incorporated. The third part was related to knowledge of the occurrence of teenage pregnancy (assessed through seven questions; these included knowledge of: the danger period or the fertility window for pregnancy, complications of teenage pregnancy, pregnancy prevention methods, information on modern contraception, type of modern contraception, double advantage of condom, where to get contraception) and history of contraception use.

### Operational definitions

Cases: Teenage girls who were pregnant at the time of the interview.

Controls: Teenage girls who had never been pregnant.

Family size: The number of family members living in a household where the study subject lived and for those who were married, the family size prior to marriage.

Parental communication on reproductive health issues: Refers to discussions, between a child (in her teenage years) and either of her parents, of topics such as menstruation and how to prevent premarital sex, HIV/AIDS and teenage pregnancy.

Knowledge of getting pregnant as a teenager: This was a dichotomous variable with "1 = good" and "0 = poor". The score of these values was calculated using the mean, with a maximum and minimum score of seven and zero, respectively.

Good knowledge of getting pregnant: Refers to those who scored above the mean from the seven knowledge-related questions.

Poor knowledge of getting pregnant: Refers to those who scored below the mean from the seven knowledge-related questions.

### Data quality control measures

Six female nurses (diploma-certified) and two male health officers working in the study area who are fluent in the local language (Tigrigna) were recruited. They were trained for one day on data collection and supervision. To ensure the clarity of the questionnaire for both data collectors and respondents, a pretest was conducted with 10% of the total sample size in a neighboring district of the study area (Enderta), with minor refinement of the study tool, like; wording and rephrasing, arrangement of questions sequence (taking most sensitive questions to the end of the questionnaire) and quantifying time required to interview respondents was made based on the results. Daily supervision was conducted by the supervisors and principal investigator to ensure the completeness and accuracy of data.

### Data management and analysis

Data were checked throughout the data collection period and up to the initiation of the analysis. Then the collected data were entered into Epi-info version 3.5.1 and transferred to SPSS version 21 for analysis. Frequencies, and proportions (tables) and means (SD), were used to represent the results of the categorical and continuous variables, respectively.

Cross tabulations were used to describe the frequencies or proportions of the study participants. Statistical significance of an association was considered at p-value ≤0.05. Multi-collinearity was checked using the variance inflation factor (VIF) test to exclude variables >10%; no variable was obtained. Bivariable logistic regression (with odds ratios and 95 percent confidence intervals) was calculated to assess the strength of the association between dependent and independent variables. Finally, multivariable logistic regression was used to assess predictors of the outcome variable. Variables with a p-value <0.25 in bivariable analysis were included in the multivariable logistic regression. Then the percentage of the model that was accurately classified was 88.2% with a Hosmer and Lemeshow test value of 0.99. In the analysis, the variability of teenage pregnancy explained by the set variables (model) ranged from 50.9 to 70.7%.

### Ethics approval and consent to participate

Ethical clearance (with reference number of ERC 0508/2015) was obtained from the ethical review board of Mekelle University, College of Health Sciences. The letters of support obtained from the Tigray Regional Health Bureau and Degua Tembien District Health Office were delivered to the concerned officials in the community. Prior to the interview, the aim of the study was explained in general, through brief information on research of adolescent health, to both the teenage girls and their parents, when applicable (i.e., if they lived in the same household) and to the teenager only if not applicable, to took verbal consent or assent. Then detailed information about the purpose of the study was given to the teenagers to obtain verbal assent or consent. A calm setting was chosen, where privacy for study subjects was ensured during the administration of the questionnaire. Confidentiality was ensured and participants’ names were not requested or recorded. Participants were also informed that they could withdraw from the interview at any time if they were not comfortable with the research questionnaire. Pregnant teenagers who had not yet started ANC were referred to health institutions.

## Result

### Socio demographic and socio-economic characteristics of study participants

The response rate of the participants in this study was 100%. The mean (±SD) age of cases and controls was 18.47 (0.72) and 17.09 (1.22) years, respectively. Three hundred and eighty seven (93.5%) study participants lived in a rural area. More than half (n = 77; 55.8%) of the cases lived with their husbands and most (n = 233; 84.4%) of the controls lived with both of their parents. Approximately half (n = 71; 51.4%) of the cases were from a family having seven or more family members, whereas a higher proportion (n = 174; 63.1%) of the controls were from a family having six or fewer family members. Regarding marital status, more than two-thirds (n = 97; 70.3%) of the cases were married; a higher proportion (n = 235; 85.1%) of the controls were single. The mean (±SD) ages of marriage and sexual debut of participants were 16.9 (1.12) and 16.77 (0.9) years respectively. In this study, 21 (15.2%) of the cases and 20 (7.2%) of the controls never received a formal education. Few cases 1 (0.7%) and controls 13 (4.7%) obtained an educational level of grade eleven or above.

In this study, 84 (60.9%) cases and 131 (47.5%) controls did not watch/listen to television (TV)/radio. Fewer cases (n = 59; 42.8%) than controls (n = 89; 32.2%) lived a distance of more than one hour from the nearby health facility (Tables 2 and 3).

**Table 2:** Socio-demographic and economic characteristics assessed as determinants of teenage pregnancy among female teenagers in Degua Tembien district, Tigray, Northern Ethiopia, 2015. (N = 414).

Variables	Teenage pregnancy		Total
	Cases	Controls	
	n (%)	n (%)	N (%)
**Age group of the participants**			
13–15 years	1 (0.7)	32 (11.6)	33 (8.0)
16–17 years	12 (8.7)	127 (46.0)	139 (33.6)
18–19 years	125 (90.6)	117 (42.4)	242 (58.5)
**Mean age** (±SD)	18.47 (0.717)	17.09 (1.2)	17.55 (1.3)
**Place of residence**			
Rural	129 (93.5)	258 (93.5)	387 (93.5)
Urban	9 (6.5)	18 (6.5)	27 (6.5)
**Marital status**			
Single	39 (28.3)	235 (85.1)	274 (66.2)
Married	97 (70.3)	34 (12.3)	131 (31.6)
Divorced	2 (1.4)	7 (2.5)	9 (2.2)
**Age at marriage or sexual debut**			
10–15 years	11 (8.0)	5 (8.1)	16 (8.0)
16–17 years	87 (63.0)	39 (62.9)	126 (63.0)
18–19 years	40 (29.0)	18 (29.0)	58 (29.0)
**Mean (±SD) age at marriage**	17 (1.12)	16.8 (0.95)	16.94 (1.1)
**Mean (±SD) age at sexual debut**	16.6 (0.9)	17.1 (0.87)	16.8 (0.9)
**Highest education level**			
None- Grade 4	58 (42.0)	46 (16.7)	104 (25.1)
Grade 5-Grade 8	68 (49.3)	145 (52.5)	213 (51.4)
Grade 9-Grade 10	11 (8.0)	72 (26.1)	83 (20.0)
Grade 11 and above	1 (0.7)	13 (4.7)	14 (3.4)
**Living arrangement**			
Both parents	51 (37)	233 (84.4)	284 (68.6)
Either parent	9 (6.5)	25 (9.1)	34 (8.2)
Husband	77 (55.8)	7 (2.5)	84 (20.3)
Alone	1 (0.7)	11 (4.0)	12 (2.9)
**Family size**			
Three or less	4 (2.9)	25 (9.1)	29 (7.0)
Four-Six	63 (45.7)	149 (54.0)	212 (51.2)
Seven and above	71 (51.4)	102 (37.0)	173 (41.8)
**Father's education level**			
None	101 (73.2)	189 (68.5)	290 (70.0)
Primary or above	27 (19.6)	70 (25.4)	97 (23.4)
Not known	10 (7.2)	17 (6.2)	27 (6.5)
**Mother's education level**			
None	119 (86.2)	234 (84.8)	353 (85.3)
Primary or above	7 (5.1)	20 (7.2)	27 (6.5)
Not known	12 (8.7)	22 (8.0)	34 (8.2)

**Table 3:** Socio-demographic and economic characteristics assessed as determinants of teenage pregnancy among female teenagers in Degua Tembien district, Tigray, Northern Ethiopia, 2015. (N = 414). . . . . . . . (continued).

Variables		Teenage	pregnancy	Total
		Cases	Controls	
		n (%)	n (%)	N (%)
**Father's occupation**				
Farmer		136 (98.6)	258 (93.5)	394 (95.2)
Other**#**		2 (1.4)	18 (6.5)	20 (4.8)
**Mother's occupation**				
Farmer		133 (96.4)	239 (86.6)	372 (89.9)
Other*		5 (3.6)	37 (13.4)	42 (10.1)
**Monthly income of family**				
below ~$25		21 (15.2)	10 (3.6)	31 (7.5)
~$ 25–50		37 (26.8)	49 (17.8)	86 (20.8)
~$ 50–100		39 (28.3)	105 (38.0)	144 (34.8)
~$ 100–150		20 (14.5)	60 (21.7)	80 (19.3)
Above ~$ 150		21 (15.2)	52 (18.8)	73 (17.6)
**Watching TV/Listening to the radio at least once per week**				
Yes		54 (39.1)	145 (52.5)	199 (48.1)
No		84 (60.9)	131 (47.5)	215 (51.9)
**Time required to reach health facility**				
One hour or below		79 (57.2)	187 (67.8)	266 (64.3)
Greater than one hour		59 (42.8)	89 (32.2)	148 (35.7)
**Time required to reach school**				
One hour or below		108 (78.6)	197 (71.4)	305 (73.7)
Greater than one hour		30 (21.7)	79 (28.6)	109 (26.3)

**#** Merchant, daily laborer, government employee.

* Housewife, merchant, daily laborer, government employee.

### Reproductive health characteristics of participants

The mean (±SD) age at menarche was 15.08 (1.06) years and was slightly younger in controls (14.9 (1.02) years) than in cases (15.4 (1.05) years. Thirty-six (29.3%) cases and 45 (17.9%) controls received information on menstruation after starting their period. One hundred and fourteen (82.6%) of the cases did not have a history of contraception use, whereas among the married/sexually active controls, 49 (79%) reported a history of contraception use. Slightly lower proportions of cases (n = 87; 63%) than controls (n = 186; 67.4%) communicated with their parents on reproductive health issues. Ninety-seven (81.5%) of the cases and 77 (29.6%) of the controls did not receive information on sex education at school. Three-fourths (76.1%) of the mothers of the cases and over one-third (42.8%) of the mothers of the controls had a history of teenage pregnancy (Table 4).

**Table 4:** Reproductive health characteristics assessed as determinants of teenage pregnancy among female teenagers in Degua Tembien district, Tigray, Northern Ethiopia, 2015. (N = 414).

Variables	Teenage pregnancy		

	Cases	Controls	Total
	n (%)	n (%)	N (%)
**Maternal history of teenage pregnancy**			
Yes	105 (76.1)	158 (57.2)	263 (63.5)
No	33 (23.9)	118 (42.8)	151 (36.5)
**Sister has history of teenage pregnancy**			
Yes	29 (21.0)	68 (24.6)	97 (23.4)
No	109 (79.0)	208 (75.4)	317 (76.6)
**Past history of contraception use**			
Yes	24 (17.4)	49 (79)	73 (36.5)
No	114 (82.4)	13 (21)	127 (63.5)
**Knowledge of occurrence of teenage pregnancy**			
Good	65 (47.1)	174 (63.0)	239 (57.7)
Poor	73 (52.9)	102 (37.0)	175 (42.3)
**Communication with parents on sexual issues**			
Yes	51 (37.0)	186 (67.4)	237 (57.2)
No	87 (63.0)	90 (32.6)	177 (42.8)
**Menarche**			
Yes	137 (99.3)	217 (78.6)	354 (85.5)
No	1 (0.7)	59 (21.4)	60 (14.5)
**Age at menarche**			
10–13 years	1 (0.7)	14 (6.5)	15 (4.2)
14–16 years	119 (86.9)	187 (86.2)	306 (86.4)
17–19 years	17 (12.4)	16 (7.3)	33 (9.3)
**Received information on menstruation**			
Yes	123 (89.1)	252 (91.3)	375 (90.6)
No	15 (10.9)	24 (8.7)	39 (9.4)
**Time received information on menstruation**			
Before menarche	87 (70.7)	207 (82.1)	294 (78.4)
After menarche	36 (29.3)	45 (17.9)	81 (21.6)
**Age at sexual debut**			
10–15 years	3 (5)	0 (0)	3 (5)
16–17 years	34 (56.7)	13 (21.7)	47 (78.3)
18–19 years	2 (3.3)	8 (13.3)	10 (16.7)
**Sex education at school**			
Received	22 (18.5)	183 (70.4)	205 (54.1)
Not received	97 (81.5)	77 (29.6)	174 (45.9)
**Past pregnancy**			
Yes	12 (8.7)	0 (0)	12 (2.9)
No	126 (91.3)	276 (100)	402 (97.1)

Among the pregnant teenagers, 57 (41.3%) reported that their pregnancies were unintended and 12 (8.7%) were pregnant for their second time. More than half (n = 66; 51.2%) of the cases started ANC after the sixteenth week of gestation while nine (6.5%) had not yet begun ANC (Table 5).

**Table 5:** Reproductive health related characteristics assessed as determinants of teenage pregnancy among pregnant female teenagers in Degua Tembien district, Tigray, Northern Ethiopia, 2015. (n = 138).

Variables	Teenage pregnancy		

	Cases	Controls	Total
	n (%)	n(%)	N (%)
**Past pregnancy**			
Intended	2 (16.7)		2 (16.7)
Unintended	10 (83.3)	NA	10 (83.3)
**Age at first pregnancy**			
10–15 years	1 (0.7)		1 (0.7)
16–17 years	24 (17.4)		24 (17.4)
18–19 years	113 (81.9)	NA	113 (81.9)
**Current pregnancy**			
Intended	81 (58.7)		81 (58.7)
Unintended	57 (41.3)	NA	57 (41.3)
**Initiation of ANC**			
Yes	129 (93.5)		129 (93.5)
No	9 (6.5)	NA	9 (6.5)
**Time of ANC registration**			
Before/at 16 weeks	63 (48.8)		63 (48.8)
After 16 weeks	66 (51.2)	NA	66 (51.2)

NA: Not applicable

### Knowledge of participants of getting pregnant

More than half (n = 73; 52.9%) of the cases had poor knowledge of the occurrence of conception compared to the controls (n = 102; 37%). One hundred and twenty-two (88.4%) of the cases and 223 (80.8%) of the controls had mentioned use of at least two types of contraceptive methods, but a higher proportion of controls (n = 216; 78.3%) than of cases (n = 87; 63.0%) were aware of the double advantage of condom use (Table 6).

**Table 6:** Pregnancy knowledge of female teenagers in Degua Tembien district, Tigray, Northern Ethiopia, 2015. (n = 414).

Variable	Teenage pregnancy		
	Cases	Controls	Total
	n (%)	n (%)	N (%)
**Knew the fertility window period for getting pregnant**			
Yes	61 (44.2)	150 (54.3)	211 (51.0)
No	77 (55.8)	126 (45.7)	203 (49.0)
**Knew at least two complications of teenage pregnancy**			
Yes	46 (33.3)	133 (48.2)	179 (43.2)
No	92 (66.7)	143 (51.8)	235 (56.8)
**Knew at least two prevention methods of teenage pregnancy**			
Yes	9 (6.5)	28 (10.1)	37 (8.9)
No	129 (93.5)	248 (89.9)	377 (91.1)
**Heard of modern contraception methods**			
Yes	126 (91.3)	229 (83.0)	355 (85.7)
No	12 (8.7)	47 (17.0)	59 (14.3)
**Can list at least two types of contraception**			
Yes	122 (88.4)	223 (80.8)	345 (83.3)
No	16 (11.6)	53 (19.2)	69 (16.7)
**Knew the double advantage of condom use**			
Yes	87 (63.0)	216 (78.3)	303 (73.2)
No	51 (37.0)	60 (21.7)	111 (26.8)
**Knew at least two sites where contraception was available**			
Yes	7 (5.1)	16 (5.8)	23 (5.6)
No	131 (94.9)	260 (94.2)	391 (94.4)
**Overall knowledge status**			
Good	65 (47.1)	174 (63.0)	239 (57.7)
Poor	73 (52.9)	102 (37.0)	175 (42.3)

### Logistic regression analysis

Variables with a p-value of <0.25 in bivariable analysis (age, marital status, educational level, family size, father's occupation, mother's occupation, monthly income, watching TV / listening to the radio, time to travel to the health facility, maternal history of teenage pregnancy, knowledge of getting pregnant and communication with parents on reproductive health issues) were fitted to the multivariable logistic regression model. Finally, family monthly income, being married, being in the age group of 18–19 years, communication with parents on sexual issues and maternal history of teenage pregnancy were predictors of teenage pregnancy. Participants with lower monthly income (below five hundred and five hundred to one thousand birr [below ~$25 and ~$25–50]) were 24 (AOR = 23.96; 95%CI 4.89–117.29) and 5 (AOR = 4.91; 95%CI 1.64–14.66) times, respectively, more likely to have teenage pregnancy than those who received more than three thousand birr (~$150) of monthly income, after adjusting for the other variables in the model. Being in the age group of 18–19 years also had a seventeen times (AOR = 16.75; 95%CI 6.45–43.47) higher odds of teenage pregnancy when compared to the 16–17 year old age group, after adjusting for other variables in the model. Teenagers who were married were 16 times (AOR = 15.91; 95%CI 7.43–34.04) more likely to have teenage pregnancy than those who were single (Tables 7 and 8).

**Table 7:** Logistic regression analysis of selected variables assessed as determinants of teenage pregnancy among female teenagers in Degua Tembien district, Tigray, Northern Ethiopia, 2015. (n = 414).

	Teenage pregnancy			
Variables	Cases	Controls	COR (95%CI)	
	n (%)	n (%)		AOR (95%CI)
**Age group of participant**				
13–15 years	1 (0.7)	32 (11.6)	0.33 (0.04–2.64)	0.43 (0.04–4.51)
16–17 years	12 (8.7)	127 (46.0)	1	1
18–19 years	125 (90.6)	117 (42.4)	11.3 (5.94–21.52)*	16.75 (6.45–43.47)**
**Marital status**				
Single	39 (28.3)	235 (85.1)	1	1
Married	97 (70.3)	34 (12.3)	17.19 (10.20–28.83)*	15.91 (7.43–34.04)**
Divorced	2 (1.4)	7 (2.5)	1.72 (0.35–8.59)	1.30 (0.16–10.73)
**Father's occupation**				
Farmer	136 (98.6)	258 (93.5)	4.46 (1.02–19.60)*	1.48 (0.20–10.89)
Others	2 (1.4)	18 (6.5)	1	1
**Mother's occupation**				
House wife	133 (96.4)	239 (86.6)	2.19 (0.81–5.94)	1.52 (0.31–7.53)
Others	5 (3.6)	37 (13.4)	1	1
**Monthly income**				
Below ~$25	21 (15.2)	10 (3.6)	5.20 (2.10–12.89)*	23.96 (4.89–117.29)**
~$ 25–50	37 (26.8)	49 (17.8)	1.87 (0.96–3.63)	4.91 (1.64–14.66)**
~$ 50–100	39 (28.3)	105 (38.0)	0.92 (0.49–1.72)	2.52 (0.90–7.06)
~$ 100–150	20 (14.5)	60 (21.7)	0.83 (0.40–1.69)	1.39 (0.46–4.15)
Above ~$ 150	21 (15.2)	52 (18.8)	1	1
**Watching TV /Listening to radio at least weekly**				
Yes	54 (39.1)	145 (52.5)	1	1
No	84 (60.9)	131 (47.5)	1.72 (1.14–2.61)*	1.42 (0.70–2.87)
**Time required to reach health facility**				
One hour or below	79 (57.2)	187 (67.8)	1	1
Greater than one hour	59 (42.8)	89 (32.2)	1.57 (1.03–2.39)*	1.014 (0.47–2.17)
**Maternal history of teenage pregnancy**				
No	33 (23.9)	118 (42.8)	1	1
Yes	105 (76.1)	58 (57.2)	2.38 (1.50–3.76)*	4.14 (1.84–9.33)**
**Knowledge of getting pregnant**				
Good	65 (47.1)	174 (63.0)	1	1
Poor	73 (52.9)	102 (37.0)	1.92 (1.27–2.90)*	1.44 (0.68–3.04)
**Communication with parents on RH issues**				
Yes	51 (37.0)	186(67.4)	1	1
No	87 (63.0)	90 (32.6)	3.53 (2.30–5.41)*	6.52 (3.12–13.64)**

*Significant at p≤0.05

** Predictors at p ≤ 0.001

**Table 8:** Logistic regression analysis of selected variables assessed as determinants of teenage pregnancy among female teenagers in Degua Tembien district, Tigray, Northern Ethiopia, 2015. (n = 414). . . . . (continued).

Variables	Teenage pregnancy		COR (95%CI)	AOR (95%CI)
	Cases	Controls		
	n (%)	n (%)		
**Highest education level**				
None- Grade 4	58 (42.0)	46 (16.7)	16.39 (2.07–129.96)*	20.24 (0.80–512.07)
Grade 4-Grade 8	68 (49.3)	145 (52.5)	6.10 (0.78–47.56)	9.08 (0.39–212.13)
Grade 9-Grade 10	11 (8.0)	72 (26.1)	1.99 (0.24–16.72)	2.59 (0.10–64.74)
Grade 11 and above	1 (0.7)	13 (4.7)	1	1
**Family size**				
Three or less	4 (2.9)	25 (9.1)	1	1
Four-Six	63 (45.7)	149 (54.0)	2.64 (0.8–7.91)	1.05 (0.26–4.20)
Seven and above	71 (51.4)	102 (37.0)	4.35 (1.45–13.05)*	0.92 (0.23–3.77)

*Significant at p≤0.05

## Discussion

This study was aimed to identify the determinants of teenage pregnancy and has revealed that being married, not communicating with parents on reproductive health issues, having a history of maternal teenage pregnancy, aged 18–19 years and lower monthly household income were the independent predictors of teenage pregnancy. Married teenagers were more likely to have teenage pregnancy compared to single teenagers. Consistent to our finding many studies conclude that in a setting where early marriage is highly prevalent, teenagers are exposed to unwanted pregnancy, unsafe abortion and STIs [4,16–21]. Marriage may force teenagers to curtail their education (23% according to our research), lose future opportunities for economic independence and reduce a women’s decision-making power [19]. However, marriage was not predictive of teenage pregnancy in the research result from a study conducted in Tanzania [22], which may be due to the background of the study participants, all of whom were enrolled in school, a factor which may allow them the opportunity to utilize health services, such as contraception. A factor which may be indirectly related to marriage—that of not living with parents—was significantly associated with teenage pregnancy in the findings of other studies [14, 23]; however our research did not show an association.

Communication with parents on reproductive health (RH) issues was also a major predictor of teenage pregnancy in our study. Teenagers who did not communicate about RH issues with their parents were more exposed to teenage pregnancy than their counter parts. In our study only 57.2% of parents of teenagers communicate with their teenagers. Lower to our study, research from Dridawa/Ethiopia reveals that only 17.9% of fathers and 25.4% of mothers were transparent and willing to discuss sexual and reproductive issues with their adolescents [24]. However, research from other African countries indicates that the proportion of parents who communicate about such matters with their teens ranges from 69% to 83% [22, 25,26], which is high compared to our study result. This may be due to differences in research methodologies and/or study populations (such as variations in the educational status of parents, place of dwelling or geographic variation). Numerous factors may explain why parents did not communicate about RH issues with their teenagers. The reasons as perceived by parents could be: teenagers are believed to be too young for RH discussions [27], the topic is considered as taboo, parents lacked knowledge of what to communicate, and the belief that teenagers had enough knowledge [28]. Communication between parents and adolescents could enable parents to address challenges of their adolescents and could help adolescents in delaying sexual activity and pregnancy [20]. Another finding from our research is that the likelihood of pregnancy of the female teenager increases with age (being in the 18–19 age groups). This finding was consistent with other researches [16,18].This is an expected result given that the proportion of women who have started their reproductive life increases with age because of longer exposure to biological and social factors, especially due to marriage.

A history of maternal teenage pregnancy represents another predictor of teenage pregnancy. Teenagers with history of maternal teenage pregnancy were more likely to have teenage pregnancy and this was consistent to other studies [29]. This finding is supported by researches which revealed the strong influence of mothers on the reproductive and sexual behaviors of their teenage daughters [24, 30, 31]. The mothers of never-pregnant teens may viewed early childbearing as more problematic, perceived older ages as appropriate for when girls first should have sex, get married, and start a family, and ascribed lower status to the parenting role than the mothers of pregnant teens [32]. However, this variable was not significantly associated with teenage pregnancy in other researches [23, 33, 34]; this inconsistency may be due to differences in methodology and sample size differences.

In our study lower family monthly income was also the predictive of teenage pregnancy. Regarding family income, the results of other studies were in line with our findings, wherein low income was predictive of teenage pregnancy [14, 20, 21, 23, 25, 35].With respect to income status of adolescents’ families; it seems plausible that the income status of their parents could affect their susceptibility to pregnancy. The findings revealed that those in the higher monthly income had the least odds of being pregnant in adolescence compared to the lowest monthly income level. A possible pathway of this influence could be that females with the lowest income tend to marry at an early age, while those with the highest income continue with their education and other career goals [4, 16, 17]. While family income was not a risk factor for teenage pregnancy in research in Sri Lanka, the study participants in that comparative analysis all had the same socioeconomic status [36].

## Conclusion

The factors associated with teenage pregnancy in our study were lower family monthly income, being married, being in the 18–19 year age group, not communicating with parents on reproductive health issues and having a maternal history of teenage pregnancy. These circumstances are multidimensional, as they are related to the individual, family, community, and the system; almost all are beyond the control of teenagers. Programs that encourage parent-teenage communication of reproductive health issues, starting from early adolescence, in order to build skills to prevent pregnancy in the late teenage years, are very important. In addition, multi-pronged activities across sectors that encourage delayed marriage and improve health service utilizations for girls are essential.

## Supporting information

S1 File This is the S1 File questionnaire. This is the S1 File questionnaire which was used to collect the data for this study.
(DOCX)

S2 File This is the S2 File copy edit. This is the S2 File on the copy edit of the manuscript for language usage, spelling, and grammar.
(DOCX)

## Abbreviations

ANC: Antenatal Care
RH: Reproductive Health
TV: Television
