# Supplementary material for: Determinants of teenage pregnancy in Degua Tembien District, Tigray, Northern Ethiopia: A community-based case-control study
Source: PLoS One. 2018 Jul 25;13(7):e0200898. doi: 10.1371/journal.pone.0200898 (PMC6059451; doi:10.1371/journal.pone.0200898)
Supplement: S1 File — This is the S1 File questionnaire which was used to collect the data for this study. (DOCX) [file pone.0200898.s001.docx]

**Mekelle University**

**Department Of Public Health**

**Structured English Version Questionnaire**

**On Determinants of Teenage Pregnancy in Degua' Tembien Distrct; Community Based Case Control Study.**

| **Section A: Biographic Information** | | | | | | | | | | |
| --- | --- | --- | --- | --- | --- | --- | --- | --- | --- | --- |
| **S.No.** | | **Demographic Questions** | **Responses and coding** | | **Skip to** | | | | **Code** | |
| 101 | | Your age at your last birthday. | in YEARS =........................ | |  | | | |  | |
| 102 | | Where do you live? | 1= Rural  2= Urban | |  | | | |  | |
| 103 | | What is your religion/faith? | 1 = Orthodox  2 = Muslim  3 = Other (specify) | |  | | | |  | |
| 104 | | What is your Ethnicity? | 1= Tigrian  2= Amhara  3= Oromo  4= Others | |  | | | |  | |
| 105 | | What is your birth order? | ................. | |  | | | |  | |
| 106 | | What is your marital status? | 1=Single  2=Married  3=Divorced  4=Widowed | | If single skip to Q N^o^ 111 | | | |  | |
| 107 | | If out of single, state the circumstances of marriage. | 1=Forced by parents  2= Voluntary  3=Failure at school  4=Pregnancy  5=Other (specify) | |  | | | |  | |
| 108 | | What was your age at marriage? | In Years  .......................... | |  | | | |  | |
| 109 | | What was the age of your husband at your marriage? | In Years  ......................... | |  | | | |  | |
| 110 | | Educational status of your husband? | 1=unable to write & read  2=Grade 1-4  3=Grade 5-8  4=Grade 9-10  5=Grade 11-12  6=College & above  7=I don't know | |  | | | |  | |
| 111 | | Have you ever been started formal education? | 1=yes  2=no | | If no skip to Q N^o^ 116 | | | |  | |
| 112 | | If Yes what is your level of education? | 1=Grade 1-4  2=Grade 5-8  3=Grade 9-10  4=Grade11-12  5=College & above | |  | | | |  | |
| 113 | | Are you currently attending school? | 1=yes  2= no | | If no skip to Q N^o^ 115 | | | |  | |
| 114 | | If yes are you participate in any of school clubs? | 1=yes  2=no | |  | | | |  | |
| 115 | | What is the reason? | 1=Educational failure  2=Lack of funds  3=Pregnancy  4=Family responsibility  5=School is not available around  6=No support from parents | |  | | | |  | |
| 116 | | With Whom do you live currently? | 1=Both parents  2=Father only  3= Mother only  4=Husband  5=Guardian/adoptive parents | |  | | | |  | |
| 117 | | If not living with both parents why? | 1=both parents dead  2=both parents working far away  3=mother working far away  4=father working far away  5=one parent dead(specify)..................  6=Divorced  7=Already married(family responsibility) | |  | | | |  | |
| 118 | | How many family members live with you? (Including yourself)( During Marriage if Married) | 1= <3  2= 4-6  3= >7  4= I don't remember | |  | | | |  | |
| 119 | | What is the educational status of your father? | 1=unable to write & read  2=Grade 1-4  3=Grade 5-8  4=Grade 9-10  5=Grade 11-12  6=College & above  7=Don't know | |  | | | |  | |
| 120 | | What is the educational status of your mother? | 1=unable to write & read  2=Grade 1-4  3=Grade 5-8  4=Grade 9-10  5=Grade 11-12  6=College & above  7=Don't know | |  | | | |  | |
| 121 | | What work does your father do? | 1=Farmer  2=Daily laborer  3=Employed  4=Merchant  5=other(Specify | |  | | | |  | |
| 122 | | What work does your mother do? | 1=Farmer  2=Housewife  3=Employed  4=Merchant  5=other(Specify | |  | | | |  | |
| 123 | | Average monthly income of the family | .........................birr | |  | | | |  | |
| 124 | | Do you have a television at your home? | 1=yes  2=no | |  | | | |  | |
| 125 | | Do you have a radio at your home? | 1=yes  2=no | |  | | | |  | |
| 126 | | If yes, do you watch television/listen radio? | 1=yes  2=no | |  | | | |  | |
| 127 | | If yes how many times per week? | 1=two times  2=three times  3=>three times  4=once | |  | | | |  | |
| 128 | | How long does it take to reach the nearest health institution on foot? | 1=<15 minutes  2=15-30 minutes  3=30-45minutes  4= 45-60 minutes  5= >l hr | |  | | | |  | |
| 129 | | How long does it take to reach the nearest school on foot? | 1=<15 minutes  2=15-30 minutes  3=30-45minutes  4= 45-60 minutes  5= >l hr | |  | | | |  | |
| **Section B: Information On Sexuality And Reproductive Health Issues** | | | | | | | | | | |
| 201 | Have you had your menstrual period? | | | 1= yes  2= no | | If no skip to Q n^o^ 206 | | | |  |
| 202 | If yes how old were you when you had the first menstrual period? | | | ..............year | |  | | | |  |
| 203 | Have you seen your menses last month? | | | 1=yes  2=no | | If no skip to Q 206 | | | |  |
| 204 | If yes, do you remember the day at your last normal menstrual period? | | | 1=yes  2=no | |  | | | |  |
| 205 | If yes when was your last menstrual period? | | | Date/month...................../......................... | |  | | | |  |
| 206 | Have you ever discuses at least with one of your parent on either of issues like, menstruation; prevent premarital sex, HIV/AIDS and teenage pregnancy in your teen age? | | | 1=Yes  2=No | | If no skip to Q 208 | | | |  |
| 207 | If yes, on what issue? | | | ..............................  ................................  ............................. | |  | | | |  |
| 208 | Have you ever been receive information about menstrual period? | | | 1= yes  2= no | | If no skip to Q 212 | | | |  |
| 209 | If yes when did you get the information? | | | 1=before menstruation  2=after menstruation | |  | | | |  |
| 210 | Who did give you the information? | | | 1=mother  2=Friend  3=teacher  4=Health professionals  5=Father 6=other (specify).................... | |  | | | |  |
| 211 | What information did you receive regarding the menstrual period? (please list) | | | 1=It is a curse  2=It is painful  3=You are ready to have a pregnancy 4=You are ready to get married  5= About hygiene  6=Avoiding sexual intercourse  7=Other (please state)......................... | |  | | | |  |
| 212 | Have you been pregnant before? | | | 1=yes  2=no | | If no skip to Q n^o^ 215 | | | |  |
| 213 | If yes, how old were you at your first pregnancy? | | | Years =................... | |  | | | |  |
| 214 | Was that a planned pregnancy? | | | 1 = yes  2= no | |  | | | |  |
| 215 | Are you currently pregnant? | | | 1=yes  2=no | | If no skip to Q 221 | | | |  |
| 216 | If yes; Have start ANC follow-up? | | | 1=yes  2=no | | If no skip to Q 219 | | | |  |
| 217 | If yes, Where is that? | | | 1=health post  2=health center  3=hospital  4=private clinic | |  | | | |  |
| 218 | When was your booking to ANC? | | | 1=Before /at 16 weeks  2=After 16 weeks | |  | | | |  |
| 219 | Is this pregnancy a planned pregnancy? | | | 1 = yes  2= no | |  | | | |  |
| 220 | If Q 216 is no; Why? | | | 1=health facility is too far  2=families not allow to do so  3=fear to health professionals  4=other (specify)..................... | |  | | | |  |
| 221 | Have you ever had sexual intercourse?(For those who are single); | | | 1=yes  2 =no | | If no skip to Q 226 | | | |  |
| 222 | At what age was your first sexual intercourse? | | | 1=Below10  2=10-12  3=13-14  4=15-17  5=18-19 | |  | | | |  |
| 223 | What led you to have sexual intercourse? | | | 1=Self desire  2=Peer pressure  3=Parent pressure  4=Pressure of partner  5 =To get pregnant  6=Violence | |  | | | |  |
| 224 | What was the age of your sexual partner at the first sexual encounter? | | | Years =................... | |  | | | |  |
| 225 | How many sexual partners have you had? | | | .................... | |  | | | |  |
| 226 | Have ever received sex education in you school?(if she has formal education) | | | 1=yes  2=no | |  | | | |  |
| 227 | Had your mother had history of teenage pregnancy? | | | 1=yes  2=no | |  | | | |  |
| 228 | Have you a sister with a history of teenage pregnancy? | | | 1=yes  2=no | |  | | | |  |
| **Section C: Knowledge on getting and prevention of teenage pregnancy** | | | | | | | | | | |
| 301 | When is a woman likely to get pregnant? | | | 1=14 days before menstruation within +3 days  2= 14 days after menstruation within +3 days  3=During menstruation  4=Anytime | | |  |  | | |
| 302 | What are the risks/dangers of indulging in sexual intercourse at an early age? | | | 1= Fail at school  2= Become pregnant  3=Contract STI/HIV/AIDS  4=Pushed out of the community | | |  |  | | |
| 303 | How would you prevent pregnancy, sexually transmitted diseases and HIV/AIDS? | | | 1=By abstaining from sexual activities  2=Using contraceptives  3=Having regular medical check-ups  4=Have only one sexual partner  5=Other (please specify) | | |  |  | | |
| 304 | In your opinion, what is the youngest age at which you consider it a risk to fall pregnant? | | | 1=20 years  2=18 years  3 =17 years  4 =16 years  5=15 years  6=14 years  7=13 years and younger | | |  |  | | |
| 305 | Have you been heard about contraceptive methods? | | | 1=yes  2=no | | |  |  | | |
| 306 | If yes mention some of the methods that you know | | | ....................................  ................................... | | |  |  | | |
| 307 | Which contraceptive method prevents pregnancy and sexually transmitted diseases? | | | 1=Intra Uterine Device  2=Pills  3 =Condom  4=Natural method/rhythm | | |  |  | | |
| 308 | Where are contraceptives available? | | | 1=Health institutions  2=Pharmacy  3= Market place  4=At schools  5=Others (specify)........... | | |  |  | | |
| 309 | Have you ever been used any contraceptives method? | | | 1=yes  2=no | | |  |  | | |
| 310 | Are you currently using contraception? | | | 1=yes  2=no | | |  |  | | |
| 311 | If yes, Who decide to use the method? | | | 1=Self only  2=with partner  3=partner only  4=other (specify)............... | | |  |  | | |
| 312 | Which method did you use? | | | 1=Pills  2=Inject able  3=Traditional contraceptives  4=Other (please state) | | |  |  | | |

**I thank you!!**

**መቐለ ዩኒቨርሲቲ**

**ኮሌጅ ጥዕና ሳይነስ**

**ክፍሊ ሕብረተሰብ ጥዕናሳይነስ**

**ሕቶታት ክፋል ብቋንቋ ትግርኛ**

| ክፍሊ”ሀ” ውልቃዊ ሓበሬታታት | | | | | | | | | |
| --- | --- | --- | --- | --- | --- | --- | --- | --- | --- |
| መለለይ ቁፅሪ | | ሕቶ | መልሲ | ሕለፍ | | | ስያሜ | | |
| 101 | | ዕድመኪ ክንደይ **እዩ** ? | ብዓመት_________ |  | | |  | | |
| 102 | | ኣበይ ትነብሪ? | 1= ገጠር  2=ከተማ |  | | |  | | |
| 103 | | ሃይማኖትኪ **እንታይ እዩ** ? | 1=ኦርቶዶክስ  2=ሙስሊም  3= ካሊእ |  | | |  | | |
| 104 | | **ብሄርኪ እንታይ እዩ?** | 1**= ትግራዋይቲ**  **2= አምሓረይቲ**  **3= ኦሮሞ**  **4= ካልእ(ይገለፅ).........** |  | | |  | | |
| 105 | | ንወለድኪ መበል ክንደይ ውላዶም ኢኺ? | .................... |  | | |  | | |
| 106 | | ኩነታት ሓዳር | 1=ዘይተመርዐወት  2=ዝተመርዐወት  3=ዝፈትሐት  4= ስብኣያ ዝሞታ | ዘይተመርዐወት እንተኾይና ናብ ሕቶ ቁፅሪ 111 ሕለፍ | | |  | | |
| 107 | | ካብ ዘይተመርዐወት ወፃኢ ንዝኾና ብኸመይ ከምዝተመርዐወት ይገለፅ? | 1=ብሓይሊ ስድራ  2=ብደሌት  3=ትምህርቲ ብምውዳቕ  4=ጥንሲ  5= ካልእ ምክንያት |  | | |  | | |
| 108 | | ክትምርዐዊ ከለኺ ዕድመኺ ክንደይ ነይሩ? | ብዓመት____________ |  | | |  | | |
| 109 | | ክትምርዐዊ ከለኺ ናይ በዓል ገዛኺ ዕድመ ክንደይ ነይሩ? | ብዓመት_____________ |  | | |  | | |
| 110 | | ናይ በዓል ገዛኺ ትምህርቲ ደረጃ? | 1= ምንባብን ምፅሓፍን ዘይኽእል  2=1-4 ክፍሊ  3=5-8 ክፍሊ  4=9-10 ክፍሊ  5=11-12 ክፍሊ  6=ኮሌጅን ልዕሊኡን  7=እይፈልጦን |  | | |  | | |
| 111 | | ስሩዕ ትምህርቲ ጀሚርኪ ዶ ነይርኪ | 1= እወ  2=ኣይፋሉን | ኣይፋሉን እንተኾይኑ ናብ ቁፅሪ 116 ሕለፍ | | |  | | |
| 112 | | መልሲ ቁፅሪ 110 እወ እንተኾይኑ ደረጃ ትምህርቲ? | 1=1-4 ክፍሊ  2=5-8 ክፍሊ  3=9-10 ክፍሊ  4=11-12 ክፍሊ  5=ኮሌጅን ልዕሊኡን |  | | |  | | |
| 113 | | ኣብዚ ሐዚ እዋን ኣብ ትምህርቲ ገበታ ዲኺ ዘለኺ? | 1= እወ  2=ኣይፋሉን | ኣይፋሉን እንተኾይኑ ናብ ቁፅሪ 115 ሕለፍ | | |  | | |
| 114 | | መልሲ ቁፅሪ 113 እወ እንተኾይኑ ኣብ ክለባት ትሳተፊ ዶ ነይርኪ? | 1= እወ  2=ኣይፋሉን |  | | |  | | |
| 115 | | መልሲ ቁፅሪ 113 ኣይፋሉን እንተኾይኑ ምክንያቱ እንታይ’ዩ? | 1= ትምህርቲ ብምውዳቕ  2=ሓገዝ ብምስኣን  3=ጥንሲ  4=ብስድራ ሓላፍነት  5=ኣብያተ ትምርቲ ኣብቲ ከባቢ ዘይምህላው  6= ወለደይ ስለዘይደገፍዎ |  | | |  | | |
| 116 | | ሐዚ ምስ መን ኢኪ ትነብሪ? | 1= ምስ ወለደይ  2=ምስ ኣቦይ ጥራሕ  3=እነይ ጥራሕ  4=ምስ በዓል ገዛይ  5=ምስ መዐበይተይ |  | | |  | | |
| 117 | | ምስ ወለድኪ እንድሕር ዘይትነብሪ ንምንታይ? | 1=ወለደይ ስለዝሞቱ  2=ወለደይ ነንበይኖም እዮም ዝነብሩ  3=እነይ ኣብ ርሑቕ እያ እትነብር |  | | |  | | |
| 118 | | ንባዕልኺ ሓዊሱ ብሓንሳብ ክንደይ ኣባላት ስድራ ትነብሩ? (ዝተመርዐወት እንተኾይና፡ ቅድሚ ምምርዓዋ) | 1= 3ተን ትሕቲኡን  2= ካብ 4-6  3= 7ተን ልዕሊኡን  4= ኣያስታውስን |  | | |  | | |
| 119 | | ኩነታት ትምህርቲ ወላዲ ኣቦኺ? | 1= ምንባብን ምፅሓፍን ዘይኽእል  2=1-4 ክፍሊ  3=5-8 ክፍሊ  4=9-10 ክፍሊ  5=11-12 ክፍሊ  6=ኮሌጅን ልዕሊኡን  7=እይፈልጦን |  | | |  | | |
| 120 | | ኩነታት ትምህርቲ ወላዲትኪ ? | 1= ምንባብን ምፅሓፍን ዘይኽእል  2=1-4 ክፍሊ  3=5-8 ክፍሊ  4=9-10 ክፍሊ  5=11-12 ክፍሊ  6=ኮሌጅን ልዕሊኡን  7=እይፈልጦን |  | | |  | | |
| 121 | | ኩነታት ስራሕ ወላዲ ኣቦኺ? | 1= ሓረስታይ  2=ማዕልታዊ ሰራሕተኛ  3= ሰራሕተኛ መንግስቲ  4= ነጋዴ  5= ካልእ (ይገለፅ) |  | | |  | | |
| 122 | | ኩነታት ስራሕ ወላዲትኪ? | 1= ሓረስታይ  2=የቤት እመቤት  3= ሰራሕተኛ መንግስቲ  4= ነጋዴ  5= ካልእ (ይገለፅ) |  | | |  | | |
| 123 | | ብማእኸላይ ወርሓዊ እቶት ስድራቤትኪ? | ...................ቅርሺ | |  | |  | | |
| 124 | | ኣብ ገዛኹም ቴሌቭዥን ኣላትኩም ዶ? | 1= እወ  2=ኣይፋሉን | |  | |  | | |
| 125 | | ኣብ ገዛኹም ራድዮ ኣላትኩም ዶ? | 1= እወ  2=ኣይፋሉን | |  | |  | | |
| 126 | | መልሲ ቁፅሪ 125/126ን እወ እንተኾይኑ፡ቴሌቭዥን /ራድዮ ትርኢ/ ታዳምፂ ዶ? | 1= እወ  2=ኣይፋሉን | |  | |  | | |
| 127 | | መልሲ ቁፅሪ 127 እወ እንተኾይኑ፡ ኣብ ሰሙን ክንደይ ግዜ? | 1= 2ተ ግዜ  2=3ተ ግዜ  3= 3ተን ልዕሊኡን  4= ሓደ ግዜ | |  | |  | | |
| 128 | | ኣብ ጥቓ ገዛኺ ናብ ዘሎ ጥዕና ትካል ብእግሪ ክንደይ ይወስድ? | 1= ትሕቲ 15 ደቒቓ  2= 15-30 ደቒቓ  3= 30-45 ደቒቓ  4= 45-60 ደቒቓ  5= ልዕሊ ሓደ ሰዓት | |  | |  | | |
| 129 | | ኣብ ጥቓ ገዛኺ ናብ ዘሎ ቤት ትምህርቲ ብእግሪ ክንደይ ይወስድ? | 1= ትሕቲ 15 ደቒቓ  2= 15-30 ደቒቓ  3= 30-45 ደቒቓ  4= 45-60 ደቒቓ  5= ልዕሊ ሓደ ሰዓት | |  | |  | | |
| **ክፍሊ “ለ” ሓበሬታ ኣብ ስነ- ፆታን ስነ-ተዋለዶን** | | | | | | | | | |
| 201 | ወርሓዊ ፅግያ ምርኣይ ጀሚርኪ ዶ? | | 1= እወ  2=ኣይፋሉን | | | ኣይፋል እንኾይኑ ናብ ቁፅሪ 206 ሕለፍ | | |  |
| 202 | መልሲ ቁፅሪ 201 እወ እንተኾይኑ፡ ዕድመኺ ክንደይ ነይሩ? | | ብዓመት___________ | | |  | | |  |
| 203 | ኣብ ዝሓለፈ ወርሒ ኸ ርኢኺ ነይርኪ ዶ? | | 1= እወ  2=ኣይፋሉን | | |  | | |  |
| 204 | መልሲ ቁፅሪ 203 እወ እወ እንተኮይኑ መአዝ ከም ዝነበረ ተስታውሲ ዶ? | | 1= እወ  2=ኣይስታውስን | | |  | | |  |
| 205 | መልሲ ቁፅሪ 204 እወ እንተኾይኑ፡ ናይ መወዳእታ ዝረአኽሉ? | | ዕለት________  ወርሒ_______  ዓ/ም________ | | |  | | |  |
| 206 | ምስ ኣዴኺወይ ኣቦኺ ብዛዕባ ስነ-ተዋልዶ(ወርሓዊ ፅግያ፤ካብ ቕድሚ መርዓፆታዊ ርክብ ምዕቓብ፤ኤች.ኣይ.ቪ ን ጥንስን) ተመያይጥኩም ዶ ትፈልጡ? | | 1= እወ  2=ኣይፋሉን | | | ኣይፋል እንተኾይኑ ቁፅሪ 208 | | |  |
| 207 | እወ እንተይኑ ኣብ ምንታይ ዛዕባ? | | ..............................  ................................  ............................. | | |  | | |  |
| 208 | ስለ ወርሓዊ ፅግያ ሓበሬታ ረኺብኪ ትፈልጢ ዶ? | | 1= እወ  2=ኣይፋሉን | | | ኣይፋል እንተኾይኑ ቁፅሪ 210 | | |  |
| 209 | መልሲ ቁፅሪ 206 እወ እንተኾይኑ፡ ሓበሬታ ዝረኸብኩሉ መዓዝ ነይሩ? | | 1= ወርሓዊ ፅግያ ምርኣይ ቅድሚ ምጅማር  2= ወርሓዊ ፅግያ ምርኣይ ድሕሪ ምጅማር | | |  | | |  |
| 210 | ሓበሬታ ዝሃበኪ መን እዩ? | | 1= ወላዲተይ  2=መሓዛይ  3= መምህረይ  4= ናይ ጥዕና በዓል ሞያ  5= ኣቦይ  6=ካልኦት ( ይገለፅ) | | |  | | |  |
| 211 | ስለ ወርሓዊ ፅግያ ዝረኸብክዮ ሓበሬታ እንታይ ዝብል ነይሩ? (ይዘርዘሩ) | | 1= ጎዳኢ ምዃኑ  2= ሕማም ምዃኑ  3= ብድሕሪኡ ጥንሲ ከጋጥም ምዃኑ  4= ንምምርዓው ድልው ምዃኑ  5= ብዛዕባ ፅሬት ኣታሓሕዝኡ  6= ፆታዊ ርክብ ክፍፀም ከም ዘይብሉ  7= ካልእ (ይገለፅ) | | | ካብ ዘይተመርዐወት ውፃኢ ንዝኾና ናብ 215 ሕለፍ | | |  |
| 212 | ቅድሚ ሐዚ ጥንሲ ኣጋጢሙኪ ይፈልጥ ዶ? | | 1= እወ  2=ኣይፋሉን | | | ኣይፋል እንተኾይኑ ናብ 215 ሕለፍ | | |  |
| 213 | መልሲ ቁፅሪ 212 እወ እንተኾይኑ፡ ዕድመኺ ኣብ ናይ ፈለማ ጥንስኺ ክንደይ ነይሩ? | | ብዓመት________ | | |  | | |  |
| 214 | ንክትጠንሲ ትልሚ ነይሩኪ ዶ? | | 1= እወ  2=ኣይፋሉን | | |  | | |  |
| 215 | ኣብዚ ሐዚ እወን ጥንሲ ኣለኪ ዶ? | | 1= እወ  2=ኣይፋሉን | | | ኣይፋል እንተኾይኑ ናብ 221 ሕለፍ | | |  |
| 216 | እወ እንተኮይኑ ቕ/ወ/ግልጋሎት ምርመራ ጀሚርኪ ዶ? | | 1=እወ  2=ኣይፋሉን | | | ኣይፋል እንተኾይኑ ናብ 219 ሕለፍ | | |  |
| 217 | እወ እንተኮይኑ ኣበይ? | | 1=አብ ጥዕና ኬላ  2=አብ ጥዕና ጣብያ  3=አብ ሆስፒታል  4=አብ ግሊ ክሊኒክ | | |  | | |  |
| 218 | ቕ/ወ/ግልጋሎት ምርመራ ዝጀመርክሉ አብ መበል ክንደይ ወርሒ ጥንሲኪ ነይሩ? | | 1= ቕድሚ 16 ሰሙን  2=ድሕሪ 16 ሰሙን | | |  | | |  |
| 219 | ንክትጠንሲ ትልሚ ነይሩኪ ዶ? | | 1= እወ  2=ኣይፋሉን | | |  | | |  |
| 220 | መልሲ ቁፅሪ 216 ኣይፋሉን እንተኮይኑ ንምንታይ? | | 1=ጥዕና ትካል ርሑቕ ሰለዝኮነ  2=ቤተሰብ ስለ ዘይፈቐዱ  3=ንጥዕና ሰብ ሞያ ስለዝፈርሖም  4=ካሊእ (ይገለፅ) | | |  | | |  |
| 221 | ፆታዊ ርክብ ፈፂምኪ ዶ ትፈልጢ? ( ንዘይተመርዐወት ጥራሕ ይምልከት) | | 1= እወ  2=ኣይፋሉን | | | ኣይፋል እንተኾይኑ ናብ 226 ሕለፍ | | |  |
| 222 | ፆታዊ ርክብ ክትጅምሪ ከለኺ ዕድመኺ ክንደይ ነይሩ? | | 1= ትሕቲ 10 ዓመት  2= 10-12 ዓመት  3= 13- 14 ዓመት  4= 15-17 ዓመት  5=18-19 ዓመት | | |  | | |  |
| 223 | ፆታዊ ርክብ ንክትጅምሪ ዝገበረኪ ምክንያት? | | 1= ዓርሰ ድሌት  2= ናይ መሓዛ ድፍኢት  3= ናይ ወለዲ ፀቕጢ  4= ናይ ፍቅረኛ ድፍኢት  5= ንምጥናስ  6= ብዓመፅ | | |  | | |  |
| 224 | ፆታዊ ርክብ እንትትጅምሪ ናይ ፍቅረኛኺ ዕድመ ክንደይ ነይሩ? | | ብዓመት_________ | | |  | | |  |
| 225 | ኣብዚ ሐዚ እዋን ክንደይ ፍቅረኛታት አለውኺ? | | ብቁፅሪ_____________ | | |  | | |  |
| 226 | ኣብ ቤት ትምህርትኺ ብዛዕባ ትምህርቲ ስነ-ፆታ ረኺብኪ ዶ ትፈልጢ?( ስሩዕ ትምህርቲ ንዘለዎን) | | 1= እወ  2=ኣይፋሉን | | |  | | |  |
| 227 | ወላዲትኪ ታሪክ ትሕቲ 20 ዓመት ጥንሲ ኣለዎን ዶ? | | 1= እወ  2=ኣይፋሉን | | |  | | |  |
| 228 | ትሕቲ 20 ዓመት እናሃለወት ዝጠነሰት ሓፍትኺ ኣላትኪ ዶ? | | 1= እወ  2=ኣይፋሉን | | |  | | |  |
| ክፍሊ “ሐ” ኩነታት ኣፍልጦ ኣብ መንቀልን መከላኸልን ትሕቲ ዕድመ ጥንሲ | | | | | | | | | |
| 301 | ሓንቲ ጓል ኣንስተይቲ ጥንሲ ካጋጥማ ዝኽእል መዓዝ እዩ? | | 1= 14 መዓልቲ ቅድሚ ወርሓዊ ፅግያ + 3 መዓልቲ  2=14 ድሕሪ ወርሓዊፅግያ + 3 መዓልቲ  3= ኣብ እዋን ወርሓዊ ፅግያ  4= ኣብ ዝኾነ እዋን | | |  | |  | |
| 302 | ምፍፃም ፆታዊ ርክብ ትሕቲ ዕድመ ንምንታይ ከቃልዕ ይኽእል? | | 1= ምውዳቕ ትምህርቲ  2= ጥንሲ  3= ንሕማማት ስነ-ተዋለዶን  4= ካብ ሕብረተሰብ ምግላል | | |  | |  | |
| 303 | ጥንስን ሕማማት ስነ-መብስዎ ከመይ ክትከላኸሊ ትኽእሊ? | | 1= ካብ ፆታዊ ርክብ ብምዕቃብ  2= መከላኸሊ ብምጥቃም  3= በብእዋኑ ጥዕናዊ ምርመራ ብምክያድ  4= ምስ ሓደ ብምውሳን  5=ካልእ (ይገለፅ) | | |  | |  | |
| 304 | ብናትኪ አታሓሳስባ ሓንቲ ጓል ንጥንሲ ክትቃለዐሉ ዕትኽእል ዘነአሰ ዓመት ክነደይ እዩ? | | 1= 20 ዓመት  2= 18ዓመት  3=17ዓመት  4=16 ዓመት  5= 15ዓመት  6=14ዓመት  7=13ዓመትን ትሕቲኡን | | |  | |  | |
| 305 | ስለ ዘመናዊ መከላኸሊ ጥንሲ ሰሚዕኺ ዶ ትፈልጢ? | | 1= እወ  2= ኣይፋሉን | | |  | |  | |
| 306 | ሕቶ ቁፀሪ 305 እወ እንተኾይኑ፡ ካብ እትፈልጥዮም ዘርዝሪ | | 1.______________  2_____________  3._____________ | | |  | |  | |
| 307 | ጥንስን ሕማማት መብስዎ ክከላኸል ዝኽእል መከላኸሊ አየናይ እዩ? | | 1=ኣብ ማህፀን ዝቕመጥ መከላኸሊ ጥንሲ  2= ክኒን  3= ኮንዶም  4= ባህላዊ መከላኸሊ | | |  | |  | |
| 308 | ዘመናዊ መከላኸሊ ጥንሲ ኣበይ ይርከቡ? | | 1= ኣብ ጥዕና ትካል  2=ቤት- መድሓኒት  3= ኣብ ዕዳጋ  4= ኣብ ቤት ትምህርቲ  5= ካልእ ( ይገለፅ) | | |  | |  | |
| 309 | ቅድሚ ሐዚ ዝኾነ ይኹን መከላኸሊ ጥንሲ ተጠቒምኪ ዶ ተፈልጢ? | | 1= እወ  2= ኣይፋሉን | | |  | |  | |
| 310 | ኣብዚ ሐዚ እዋን መከላኸሊ ጥንሲ ትጥቀሚ ዶ? | | 1= እወ  2= ኣይፋሉን | | |  | |  | |
| 311 | መልሲ ቁፅሪ 310 እወ እንተኾይኑ፡ ንክትጥቀሚ ዝወሰነ መን ነይሩ? | | 1= ባዕለይ  2=መፃምድተይ/ፍቅረኛይ ተስማዕሚዐ  3=መፃምድተይ/ፍቅረኛይ ጥራሕ  4=ካልእ ( ይገለፅ) | | |  | |  | |
| 312 | እንታይ ዓይነት ኢኺ እትጥቀሚ? | | 1= ክኒን  2= መርፍእ  3=ባህላዊ መከላኸሊ  4= ካልእ ( ይገለፅ) | | |  | |  | |

ብጣዕሚ እየ ዘመስግን!!
