# Supplementary material for: Determinants of teenage pregnancy in Degua Tembien District, Tigray, Northern Ethiopia: A community-based case-control study
Source: PLoS One. 2018 Jul 25;13(7):e0200898. doi: 10.1371/journal.pone.0200898 (PMC6059451; doi:10.1371/journal.pone.0200898)
Supplement: S2 File — This is the S2 File on the copy edit of the manuscript for language usage, spelling, and grammar. (DOCX) [file pone.0200898.s002.docx]

**Determinants of teenage pregnancy in Degua Tembien District, Tigray, Northern Ethiopia: A community-based case-control study**

**Authors**

**Authors:**

**Brhane Ayele^1*^, Tesfay Gebregzabher^2^, Tesfay Hailu^2^, Belete Assefa^2^**

**^1^Public Health Department, Public Health Emergency Management Directorate, Tigray Health Research Institute, Mekelle, Ethiopia.**

**^2^School of Public Health, Mekelle University, Mekelle, Ethiopia.**

***Corresponding author**

E-mail: [brhane3127@gmail.com](mailto:brhane3127@gmail.com) (BA)

**Abstract**

**Background:** Approximately 16 million teenagers aged 15-19 years and 2 million teenagers under the age of 15 years give birth annually, with 95% of these births occurring in developing countries. Ethiopia has one of the highest teenage fertility rates in Sub-Saharan Africa, however determinants of teenage pregnancy are not well studied. Therefore, this study aimed to identify determinants of teenage pregnancy among female teenagers in Degua Tembien district, Tigray, Northern Ethiopia, in 2015.

**Methods:** A community-based case-control study was conducted in Degua' Tembien district from February 01, 2015 to March 15, 2015 with a randomly selected total sample size of 414 females (with a ratio of 1:2 case to control, 138 and 276 respectively). Data were entered into Epi-Info and analyzed using SPSS software. Multivariable logistic regression was used to assess predictors of the outcome variable; variables with a p-value <0.25 in bivariable analysis were included in the model. Statistically significance was considered at a p-value <0.05 in both bivariable and multivariable logistic regression analyses.

**Result:** The mean ages (plus or minus one standard deviation (+SD)) of cases and controls were 18.47 (0.72) and 17.09 (1.2) years, respectively. After adjustment for other variables, predictors of teenage pregnancy included: lower monthly income (below five hundred and five hundred to one thousand birr [below ~$25 and ~$25-50]) (adjusted odds ratio (AOR)=23.96; 95% confidence interval (95%CI) 4.89-117.29 and AOR=4.91; 95%CI 1.64-14.66, respectively); aged 18-19 years (AOR=16.75; 95%CI 6.45-43.47); being married (AOR=15.91; 95%CI 7.43-34.04); not communicating with parents on reproductive health issues (AOR=6.52; 95%CI 3.12-13.64) and having a history of maternal teenage pregnancy (AOR=4.14; 95%CI 1.84-9.33).

**Conclusion:** Programs that encourage parent-teenage communication of reproductive health issues, starting from early adolescence, in order to build skills to prevent pregnancy in the late teenage years, are very important. In addition, multi-pronged activities across sectors that encourage delayed marriage and improve health service utilizations for girls are essential.

**Key words:**Determinants, pregnancy,Teenagers, Adolescents.

**Introduction**

Approximately 16 million adolescent girls aged 15-19 years and 2 million adolescents under the age of 15 years give birth annually. These births constitute roughly 11% of all births worldwide; nearly 95% occur in developing countries. The proportion of adolescents giving birth ranged from 2% in China, to 18% in Latin America and the Caribbean, to more than 50% in sub-Saharan Africa[1]. Half of all adolescent births occur in just seven countries: Bangladesh, Brazil, the Democratic Republic of Congo, Ethiopia, Nigeria, India and the United States [2]. Each year, births to adolescent girls aged 15 to 19 years account for 16% of all births in sub-Saharan Africa [3].

In developing countries, approximately 90% of births to adolescents occur within marriage due to the high rate of early marriage [2]. Early marriage can have various implications for reproductive health. Married adolescents have earlier and higher fertility, poor fertility outcomes, inadequate child spacing, and lower contraceptive use compared to young adults [4]. Women under 20 years of age face a higher risk of obstructed labor, which can cause obstetric fistula and its complications, unsafe abortion and STIs, including HIV/AIDS [5].

Teenage pregnancy is also highly associated with social stigma, stillbirth, low birth weight and maternal death. Furthermore, the complete lack of access to health care, absence of skilled delivery services, or delayed entry into antenatal care (ANC) deprive both the teenage mother and her offspring of basic health care services [6]. Giving birth as a teenager leads to having more children than women who start childbearing activities after their teenage years. The outcome of such phenomenon contributes to high population growth. In addition, the likelihood of access to education of children born to teenagers is rare. Among those who gain access to education, frequent interruptions and absenteeism may lead to be poor academic results; consequently, these children may face unemployment when they grow up and be less physically fit for labor at a later age. Female teenagers’ may be particularly at risk for sexual harassment and rape due to poor physical fitness. Furthermore, their inability to negotiate safer and delayed sexual debut contributes to early pregnancy, as experienced by their mothers. This process continues repeatedly, which contributes to the perpetuation of the vicious cycle of poverty[7-10].

The government of Ethiopia has taken many measures to reduce teenage pregnancy and its consequences. Some of the measures taken thus far include amending and/or implementing: a law against early marriage; a national adolescent and youth reproductive health strategy; the legalization of abortion; a youth and HIV/AIDS policy; and community mobilization against harmful traditional practices, including the association of such practices with the new criminal law. However, Ethiopia has one of the highest adolescent fertility rates in sub-Saharan Africa – 72 births for every 1,000 young women aged 15-19 years[11,12]. Research on outcomes of teenage pregnancy reveal evidence of adverse effects for the teenage mother, her offspring and society as a whole[5-12]. Most of this research has compared pregnant teenagers or teenage mothers to pregnant adults or adult mothers. While this research is important, to identify the determinants of teenage pregnancy, both the control group and the cases should be teenagers. Research of determinates of teenage pregnancy in Ethiopia is extremely limited. While at least one study has associated teenage pregnancy with early marriage[12], this single factor alone can not constitute all determinants of teenage pregnancy . The dearth of information on determinants of teenage pregnancy is particularly pronounced in Tigray regional state, supporting the need for further evidence.

Therefore, this study aimed to determine the factors contributing to teenage pregnancy to help policy makers, program managers and health care authorities with better decision making in planning and problem solving.

**Methods**

**Study area and period**

This study was conducted in Degua' Tembien district from February 01 to March 15, 2015. Degua' Tembien is found 831 kilometers north of Addis Ababa, the capital city of Ethiopia, and 50 kilometers west of Mekelle, the capital city of Tigray regional state. The estimated total population of the district is 138,334, based on the 2007 census; in 2014/2015, 67,369 (48.7%) were male and 70, 965 (51.3%) female. The district has one newly-upgraded primary hospital, five health centers, 24 health posts, two private drug shops, sixty-one primary schools, one high school and one preparatory school. The primary hospital is the only health facility providing youth-friendly services among all of the health facilities in the district. Degua’ Tembien is one of the least performing districts in terms of family planning and institutional delivery utilization. The contraception acceptance and skilled attendance rates were reported as 47.8% and 35.7%, respectively, which are much lower than the regional averages of 54.8% and 50%, respectively. Moreover, Degua' Tembien is among the districts with the highest stillbirth rate and neonatal death rate, at 2.2% and 0.62%, respectively[13]. Though not documented, from observation, the district has a high rate of teenage pregnancy.

**Study design**

A community-based case-control study design was used to assess determinants of teenage pregnancy.

**Source population**

Female teenagers in Degua Tembien district constituted the source population.

**Study population**

Female teenagers registered in the pre-survey in the study area (290 pregnant and 1608 non-pregnant teenagers) were eligible for participation.

**Study unit**

Actual participants included 138 pregnant and 276 non-pregnant teenagers in Degua Tembien district.

**Sample size calculation**

n_1_=$\frac{\left( Z1-\alpha/2+Z1-\beta\right)^{2}2P\left( 1-P \right)}{\left( p1-p2 \right)^{2}}\times\frac{c+1}{2c}$

= $\frac{\left( Z1-\alpha/2+Z1-\beta\right)^{2}P\left( 1-P \right)}{\left( p1-p2 \right)^{2}}\times\frac{c+1}{c}$

n_2=_ cxn_1_

Where:

Z1- α/2 (95%CI) =1.96

P=Average of P_1_ and P_2_

Z1- 𝛽 (80% power) =0.84

P1= Proportion of exposure among cases P_2_= Proportion of exposure among controls C= Ratio of controls to cases=2:1 n_1_=Number of casesn_2_=Number of controls

Based on research conducted in South Africa on risk factors for teenage pregnancy, living with both parents was less common among pregnant teenagers. In that research, a higher proportion (50.7%) of controls was reported to "live with both parents" compared to cases (35.6%)[6]. Sample size was calculated with an assumption of 95%CI, 80% power and a 10% contingency for the non-response rate, using predictor variables such as: forced sexual debut, not living with biological father, being from a non nuclear family and living with both parents. Of all the variables, the latter—“living with both parents”—was selected as it gives the highest sample size compared to the other variables. Finally, a total sample size of 414, with 138 cases and 276 controls, was utilized in the study (Table 1).

**Sampling methods and procedures**

Cases were pregnant teenagers (between the ages of 13-19 years) at the time of interview and controls were those who had never been pregnant, from the same age group as the cases. Two weeks before the data collection period, a pre-survey was conducted by house-to-house visit to develop a sampling frame for both cases and controls. Finally, cases were selected randomly using a computer-based program (Open-Epi random program) from the already developed sampling frame, after proportional allocation to the health institutions (one primary hospital and five health centers), based on the existence of cases. Two controls were also selected randomly for each case from the same "kebelle" (district) using the identical random program.

**Data Collection techniques and tools**

A thorough literature review of risk factors for teenage pregnancy was conducted prior to the development of a questionnaire to identify determinants of teenage pregnancy. Determinants previously identified by other researchers and potential determinants for the local setting were recognized using information on reports from the regional health bureau and district health office. A rough assessment and observation of reproductive health services were conducted. Then a structured interviewer-administered questionnaire on potential determinants of teenage pregnancy was developed in English and translated to the local language of the study area (Tigrigna) prior to the start of the fieldwork. The questionnaire had three parts. The first part was related to the socio-demographic and socio-economic characteristics of respondents, such as: marital status, living arrangements, family size, family income and educational status of both the study subjects and their parents. The second part was comprised of topics related to sexuality and reproductive health, such as: age at menarche, family history of teenage pregnancy, communication with parents on reproductive health issues, and past and current pregnancy status. In addition to this, questions related to the details of pregnancy, such as: parity, planning of the current pregnancy, and health service utilization (e.g., ANC) were also incorporated. The third part was related to knowledge of the occurrence of teenage pregnancy, assessed through seven questions; these included knowledge of: the danger period, complications of teenage pregnancy, prevention methods, information on modern contraception, type of modern contraception, double advantage of condom, where to get contraception) and history of contraception use.

**Operational definitions**

Cases: Teenage girls who were pregnant at the time of the interview.

Controls: Teenage girls who had never been pregnant.

Family size: The number of family members living in a household where the study subject lived. For those who were married, the family size prior to marriage.

Parental communication on reproductive health issues: Refers to discussions, between a child (in her teenage years) and either of her parents, of topics such as menstruation and how to prevent premarital sex, HIV/AIDS and teenage pregnancy.

Knowledge of getting pregnant as a teenager: This was a dichotomous variable with "1=good" and "0=poor". The score of these values was calculated using the mean, with a maximum and minimum score of seven and zero, respectively.

Good knowledge of getting pregnant: Refers to those who scored above the mean from the seven knowledge-related questions.

Poor knowledge of getting pregnant: Refers to those who scored below the mean from the seven knowledge-related questions.

**Data quality control measures**

Six female nurses (diploma-certified) and two male health officers working in the study area who are fluent in the local language (Tigrigna) were recruited. They were trained for one day on data collection and supervision. To ensure the clarity of the questionnaire for both data collectors and respondents, a pretest was conducted with 10% of the total sample size in a neighboring district of the study area (Enderta) , with further refinement of the study tool based on the results. Daily supervision was conducted by the supervisors and principal investigator to ensure the completeness and accuracy of data.

**Data management and analysis**

Data were checked throughout the data collection period and up to the initiation of the analysis. Then the collected data were entered into Epi-info version 3.5.1 and transferred to SPSS version 21 for analysis. Frequencies, and proportions (tables) and means (SD), were used to represent the results of the categorical and continuous variables, respectively.

Cross tabulations were used to describe the frequencies or proportions of the study participants. Statistical significance of an association was considered at p-value <0.05. Multi-collinearity was checked using the variance inflation factor (VIF) test to exclude variables >10%; no variable was obtained. Bivariable logistic regression (with odds ratios and 95 percent confidence intervals) was calculated to assess the strength of the association between dependent and independent variables. Finally, multivariable logistic regression was used to assess predictors of the outcome variable. Variables with a p-value <0.25 in bivariable analysis were included in the multivariable logistic regression. Then the percentage of the model that was accurately classified was 88.2% with a Hosmer and Lemeshow test value of 0.99. In the analysis, the variability of teenage pregnancy explained by the set variables (model) ranged from 50.9 to 70.7%.

**Result**

**Socio demographic and socio-economic characteristics of study participants**

The response rate of the participants in this study was 100%. The mean (+SD) age of cases and controls was 18.47 (0.72) and 17.09 (1.22) years, respectively. Three hundred and eighty seven (93.5%) study participants lived in a rural area. More than half (n=77; 55.8%) of the cases lived with their husbands and most (n=233; 84.4%) of the controls lived with both of their parents. Approximately half (n=71; 51.4%) of the cases were from a family having seven or more family members, whereas a higher proportion (n=174; 63.1%) of the controls were from a family having six or fewer family members. Regarding marital status, more than two-thirds (n=97; 70.3%) of the cases were married; a higher proportion (n=235; 85.1%) of the controls were single. The mean (+SD) ages of marriage and sexual debut of participants were 16.9 (1.12) and 16.77 (0.9) years respectively .In this study, 21 (15.2%) of the cases and 20 (7.2%) of the controls never received a formal education. Few cases 1 (0.7%) and controls 13 (4.7%) obtained an educational level of grade eleven or above.

In this study, 84 (60.9%) cases and 131 (47.5%) controls did not watch/listen to television (TV)/radio. Fewer cases (n=59; 42.8%) than controls (n=89; 32.2%) lived a distance of more than one hour from the nearby health facility (Tables 2 and 3).

**Reproductive health characteristics of participants**

The mean (+SD) age at menarche was 15.08 (1.06) years and was slightly younger in controls (14.9 (1.02) years) than in cases (15.4 (1.05) years. Thirty-six (29.3%) cases and 45 (17.9%) controls received information on menstruation after starting their period. One hundred and fourteen (82.6%) of the cases did not have a history of contraception use, whereas among the married/sexually active controls, 49 (79%) reported a history of contraception use. Similar proportions of cases (n=87; 63%) and controls (n=186; 67.4%) communicated with their parents on reproductive health issues. Ninety-seven (81.5%) of the cases and 77 (29.6%) of the controls did not receive information on sex education at school. Three-fourths (76.1%) of the mothers of the cases and over one-third (42.8%) of the mothers of the controls had a history of teenage pregnancy prior to the data collection of this study (Table 4).

Among the pregnant teenagers, 57 (41.3%) reported that their pregnancies were unintended and 12 (8.7%) were pregnant for their second time during the data collection period. More than half (n=66; 51.2%) of the cases started ANC after the sixteenth week of gestation while nine (6.5%) had not yet begun ANC (Table 5).

**Knowledge of participants of getting pregnant**

More than half (n=73; 52.9%) of the cases had poor knowledge of the occurrence of conception compared to the controls (n=102; 37%). One hundred and twenty-two (88.4%) of the cases and 223 (80.8%) of the controls had mentioned use of at least two types of contraceptive methods, but a higher proportion of controls (n=216; 78.3%) than of cases (n=87; 63.0%) were aware of the double advantage of condom use (Table 6).

**Logistic regression analysis**

Variables with a p-value of <0.25 in bivariable analysis (age, marital status, educational level, family size, father's occupation, mother's occupation, monthly income, watching TV / listening to the radio, time to travel to the health facility, maternal history of teenage pregnancy, knowledge of getting pregnant and communication with parents on reproductive health issues) were fitted to the multivariable logistic regression model. Finally, family monthly income, being married, being in the age group of 18-19 years, communication with parents on sexual issues and maternal history of teenage pregnancy were predictors of teenage pregnancy. Participants with lower monthly income (below five hundred and five hundred to one thousand birr [below ~$25 and ~$25-50]) were 24 (AOR= 23.96; 95%CI 4.89-117.29) and 5 (AOR= 4.91; 95%CI 1.64-14.66) times, respectively, more likely to have teenage pregnancy than those who received more than three thousand birr (~$150) of monthly income, after adjusting for the other variables in the model. Being in the age group of 18-19 years also had a seventeen times (AOR= 16.75; 95%CI 6.45-43.47) higher odds of teenage pregnancy when compared to the 16-17 year old age group, after adjusting for other variables in the model. Teenagers who were married were 16 times (AOR= 15.91; 95%CI 7.43-34.04) more likely to have teenage pregnancy than those who were single (Tables 7 and 8).

**Discussion**

Many studies conclude that in a setting where early marriage is highly prevalent, teenagers are exposed to unwanted pregnancy, unsafe abortion and STIs. Consistent with other studies, we found that marriage was a predictor of teenage pregnancy[6,14-17]. Marriage may force teenagers to curtail their education (23% according to our research), lose future opportunities for economic independence and reduce a women’s decision-making power. However, marriage was not predictive of teenage pregnancy in the research result from a study conducted inTanzania[20], which may be due to the background of the study participants, all of whom were enrolled in school, a factor which may allow them the opportunity to utilize health services, such as contraception. A factor which may be indirectly related to marriage—that of not living with parents—was significantly associated with teenage pregnancy in the findings of other studies[18, 19], however our research did not show an association.

Communication with parents on reproductive health (RH) issues was also a major predictor of teenage pregnancy in our study. Numerous factors may explain why parents did not communicate about RH issues with their teenagers (only 57.2% in our study). The perceived reasons were: a) Teenagers were believed to be too young for RH discussions, b) The topic was considered as taboo, c) Parents lacked knowledge of what to communicate, and d) The belief that teenagers had enough knowledge[21]. Similar to our study, research from Dridawa reveals that only 17.9% of fathers and 25.4% of mothers were transparent and willing to discuss sexual and reproductive issues with their adolescents [22].However, research from other African countries indicates that the proportion of parents who communicate about such matters with their teens ranges from 69% in South Africa to 83% in Tanzania[20, 23], which is high compared to our study (57.2%). This may be due to differences in research methodologies and/or study populations (such as variations in the educational status of parents, place of dwelling or geographic variation). While the topic is debated, many researchers have found that parental communication with teenagers is significantly associated with teenage pregnancy[24, 25]. A history of maternal teenage pregnancy represents another predictor of teenage pregnancy. This is supported by research which revealed the strong influence of mothers on the reproductive and sexual behaviors of their teenage daughters[22, 26, 27]. In our study, maternal history of teenage pregnancy was predictive of daughter teenage pregnancy; other studies [19, 28] using the same study design have also found this association. However, this variable was not significantly associated with teenage pregnancy in research conducted in Turkey[29]; this inconsistency may be due to differences in methodology, as the study design in Turkey was cross-sectional.

Regarding family income, the results of other studies were in line with our findings, wherein low income was predictive of teenage pregnancy[18, 19, 23, 30].With respect to income status of adolescents’ families, it seems plausible that the income status of their parents could affect their susceptibility to pregnancy. The findings revealed that those in the higher monthly income had the least odds of being pregnant in adolescence compared to the lowest monthly income level. A possible pathway of this influence could be that females with the lowest income tend to marry at an early age, while those with the highest income continue with their education and other career goals[6, 14, 15]. While family income was not a risk factor for teenage pregnancy in research in Sri Lanka, the study participants in that comparative analysis all had the same socioeconomic status [24].

Another finding from our research is that the likelihood of pregnancy of the female teenager increases with age (being in the 18-19 age group). This finding was consistent with other research[14,16].This is an expected result given that the proportion of women who have started their reproductive life increases with age because of longer exposure to biological and social factors, especially due to marriage, which is consistent with the findings of other studies.

**Conclusion**

The factors associated with teenage pregnancy in our study were lower family monthly income, being married, being in the 18-19 year age group, not communicating with parents on reproductive health issues and having a maternal history of teenage pregnancy. These circumstances are multidimensional, as they are related to the individual, family, community, and the system; almost all are beyond the control of teenagers. Programs that encourage parent-teenage communication of reproductive health issues, starting from early adolescence, in order to build skills to prevent pregnancy in the late teenage years, are very important. In addition, multi-pronged activities across sectors that encourage delayed marriage and improve health service utilizations for girls are essential.

**List of abbreviations**

ANC Antenatal Care

ETB Ethiopian Birr

RH Reproductive Health

TV Television

**Ethics approval and consent to participate**

Ethical clearance was obtained from the ethical review board of Mekelle University, College of Health Sciences. The letters of support obtained from the Tigray Regional Health Bureau and Degua Tembien District Health Office were delivered to the concerned officials in the community. Prior to data collection, the aim of the study was explained in general, through brief information on research of adolescent health, to both the teenage girls and their parents, when applicable (i.e., if they lived in the same household). At the end of the interview, detailed information about the purpose of the study was given to the teenagers. A calm setting was chosen, where privacy for study subjects was ensured during the administration of the questionnaire. Confidentiality was ensured and participants’ names were not requested or recorded. Participants were also informed that they could withdraw from the interview at any time if they were not comfortable with the research questionnaire. Pregnant teenagers who had not yet started ANC were referred to health institutions.

**Authors' contributions**

AB^1^ Contributed to the study conception, design, data collection, analysis, interpretation and write up of the manuscript.

GT^2^ Contributed to the study conception, providing scientific advice on: the design of the study, data analysis and the manuscript.

HT^3^ Contributed to the design, review of the analysis, and the revision, interpretation and write up of the manuscript.

AB^4^ Contributed to the revision of the manuscript.

All authors read and approved the final manuscript.

**Authors' information**

1. Brhane Ayele (MPH): Expert at Tigray Public Health Research Institute, Tigray, Ethiopia.

2. Tesfay Gebrehiwot (PhD): Assistant Professor at Mekelle University, Tigray, Ethiopia.

3. Tesfay Hailu (MPH): Lecturer at Mekelle University, Tigray, Ethiopia.

4. Belete Assefa (MD, MPH): Assistant Professor at Mekelle University, Tigray, Ethiopia.

**Acknowledgement**

The authors would like to thank the distinguished families, friends, study participants, data collectors and supervisors for the contributions they made for the completion of this study.

**References**

1. World Health Organization (WHO). Why is giving special attention to adolescents important for achieving Millennium Development Goal 5. Making Pregnancy Safer; 2008.

2.Mangiaterra V, Pendse R, McClure K, Rosen J. Adolescent pregnancy. Make Pregnancy Safer ;2008.

3. United Nation Funds for Population Agency(UNFPA). Status report adolescents and young people in Sub-Saharan Africa opportunities and challenges; 2012.

4.Sarkar P. Determinants and effects of early marriage in Bangladesh.Journal of applied science. 2009; 4(5):178-84.

5.Berglas N, Brindis C, Cohen J. Adolescent pregnancy and childbearing in California.California Research Bureau; 2003.

6.Vundule C, Maforah F, Jewkes R, Ordaan E. Risk for Teenage pregnancy among sexually active black adolescents in Cape town.Africa journal.2001; 91(1).

7.Unicef Fact Sheet. Young people and family planning: Teenage pregnancy;2008.

8. Mekonnen WA. Differentials of early teenage pregnancy in Ethiopia, 2000, and 2005: ICF International Calverton, Maryland, USA;2013.

9. World Health Organization (WHO). Adolescent pregnancy. Geneva, department of child and adolescent health and development;2004.

10. World Health Organization (WHO).Early marriages, adolescent and young pregnancies; 2012.

11.Perezieto P, Tefera B. Social justice for adolescent girls in Ethiopia: Tackling lost potential; 2013.

12. Jones N, Tefera B, Stephenson J, Gupta T, Pereznieto P, Emire G, et al. Early marriage and education the complex role of social norms in shaping Ethiopian adolescent girls' lives: Overseas Development Institute ;2014.

13.Tigray Regional Health Bureau(TRHB). Tigray regional health bureau annual profile; 2013.

14. Gideon R. Factors associated with adolescent pregnancy and fertility in Uganda: analysis of the 2011 demographic and health survey data. Social Sciences; 2013; 2(1): 7-13.

15. Isa I, Gani O. Socio-demographic determinants of teenage pregnancy in the Niger Delta of Nigeria. Open Journal of Obstetrics and Gynecology; 2012 2:239-43.

16. Alemayehu T, Haider J, Habte D. Determinants of adolescent fertility in Ethiopia: Ethiopian journal health development; 2010; 24(1):30-8.

17.Beguy D, Ndugwa R, Kabiru W. Entry in to motherhood among adolescent girls in two informal settlements in Nairobi,Kenya. Journal of biosocial science; 2013; 45(06):721 - 42.

18. Omar K, Hasim S, Muhammad A, Jaffar A, Hashim M, Siraj H. Adolescent pregnancy outcomes and risk factors in Malaysia: International Journal of Gynecology and Obstetrics;2010:220–3.

19. Goicolea I, Wulff M, Öhman A, Sebastian M. Risk factors for pregnancy among adolescent girls in Ecuador’s Amazon basin: Pan Am journal public health;2009;26(3):221-8.

20. Peter N. Factors associated with adolescent pregnancies among secondary school students a study from Tanga-Tanzania: Tanzania Medical Students’ Association;2008/2009.

21. Hyde A, Carney M, Drennan J, Butler M, Lohan M, Howlett E. Parents’ Approaches to Educating their Pre-adolescent and Adolescent Children about Sexuality: University College Dublin and Queen’s University;2009.

22.Ayalew M, Mengistie B, Semahegn A. Adolescent - parent communication on sexual and reproductive health issues among high school students in Dire Dawa, Eastern Ethiopia: Reproductive Health;2014; 11(77).

23.Mchunu G, Peltzer K, Tutshana B, Seutlwadi L. Adolescent pregnancy and associated factors in South African youth: African Health Sciences; 2012;12(4):426 - 34.

24.Dulitha F, Nalika G, Upul S, Chrishantha M, Alwis RD, Hemantha S, et al. Risk factors for teenage pregnancies in Sri Lanka: Journal of health science;2013;7(3 ).

25.Bushaija E, Sunday F , Asingizwe D, Olayo R , Abong’o B. Factors that hinder parents from the Communicating of sexual matters with adolescents in Rwanda: Rwanda journal health science;2013;2(2).

26. Woodward J, Horwood J, Fergusson M. Teenage pregnancy cause for concern: New Zealand medical journal; 2001; 114(1135):301-3.

27.Wamoyi J, Fenwick A, Urassa M, Zaba B, Stones W. Parent-child communication about sexual and reproductive health in rural Tanzania: Implications for young people's sexual health interventions. Reproductive-health-journal;2010;7(6).

28. Patricia L. Barbara T. Reyes J, Emily J. Association between adolescent pregnancies a family history of teenage births.Guttmacher Institute; 2007; 39(2).

29.Oner S, Yapici G, Kurt O, Sasmaz T, Bugdayci R. The sociodemographic factors related with the adolescent pregnancy. Asian Pacific Journal of Reproduction. 2012;1(2):135-41.

30.Raghav R, Chaudhari N, BhutaniT, Bamane S. Teenage Pregnancy aproduct of various socialand health factors. Indian Medical Gazette;2013.

Table 1**: Sample size calculation for the predictors of teenage pregnancy among female teenagers in Degua Tembien District, Tigray, Northern Ethiopia based on significant factors from other studies.**

| **Significant** | **Citation** | **CI** | **Power** | **Case:** | **Proportion of exposure** | | **OR** | **Samples size including 10%** | | |
| --- | --- | --- | --- | --- | --- | --- | --- | --- | --- | --- |
| **predictors** |  |  |  | **Control** | **Case** | **Control** |  | **Case** | **Control** | **Total** |
| 1.Household not a nuclear | (6) | 95 | 80 | 1:2 | 0.845 | 0.522 | 4.00 | 38 | 76 | 114 |
| family |  |  |  |  |  |  |  |  |  |  |
| 2.Not living with biological | (6) | 95 | 80 | 1:2 | 0.744 | 0.521 | 2.62 | 62 | 124 | 186 |
| father |  |  |  |  |  |  |  |  |  |  |
| 3.Biological parents live | (6) | 95 | 80 | 1:2 | 0.356 | 0.507 | 0.55 | 138 | 276 | 414 |
| together |  |  |  |  |  |  |  |  |  |  |
| 4.Sexual debut | (6) | 95 | 80 | 1:2 | 0.319 | 0.181 | 2.35 | 123 | 246 | 369 |
| forced or raped |  |  |  |  |  |  |  |  |  |  |
| 5.Education level of teenager: primary school and below | (26) | 95 | 80 | 1:2 | 0.904 | 0.791 | 1.95 | 136 | 271 | 407 |

* Therefore the total sample size was 414 (cases (n_1_) =138 and controls (n_2_) =276).

Table 2: **Sociodemographic and economic characteristics assessed as determinants of teenage pregnancy among female teenagers in Degua Tembien District, Tigray, Northern Ethiopia, 2015. (N=414)**

| **Variables** | **Teenage pregnancy** | | **Total** |
| --- | --- | --- | --- |
|  | **Cases** | **Controls** |  |
|  | **n (%)** | **n (%)** | **N (%)** |
| **Age group of the participants** |  |  |  |
| 13-15 years | 1(0.7) | 32(11.6) | 33(8.0) |
| 16-17 years | 12(8.7) | 127(46.0) | 139(33.6) |
| 18-19 years | 125(90.6) | 117(42.4) | 242(58.5) |
| **Mean age** (+SD) | 18.47(0.717) | 17.09(1.2) | 17.55(1.3) |
| **Place of residence** |  |  |  |
| Rural | 129(93.5) | 258(93.5) | 387(93.5) |
| Urban | 9(6.5) | 18(6.5) | 27(6.5) |
| **Marital status** |  |  |  |
| Single | 39(28.3) | 235(85.1) | 274(66.2) |
| Married | 97(70.3) | 34(12.3) | 131(31.6) |
| Divorced | 2(1.4) | 7(2.5) | 9(2.2) |
| **Age at marriage or sexual debut** |  |  |  |
| 10-15 years | 11(8.0) | 5(8.1) | 16(8.0) |
| 16-17 years | 87(63.0) | 39(62.9) | 126(63.0) |
| 18-19 years | 40(29.0) | 18(29.0) | 58(29.0) |
| **Mean (+SD) age at marriage** | 17(1.12) | 16.8(0.95) | 16.94(1.1) |
| **Mean (+SD) age at sexual debut** | 16.6(0.9) | 17.1(0.87) | 16.8(0.9) |
| **Highest education level** |  |  |  |
| None- Grade 4 | 58(42.0) | 46(16.7) | 104(25.1) |
| Grade 5-Grade 8 | 68(49.3) | 145(52.5) | 213(51.4) |
| Grade 9-Grade 10 | 11(8.0) | 72(26.1) | 83(20.0) |
| Grade 11 and above | 1(0.7) | 13(4.7) | 14(3.4) |
| **Living arrangement** |  |  |  |
| Both parents | 51(37) | 233(84.4) | 284(68.6) |
| Either parent | 9(6.5) | 25(9.1) | 34(8.2) |
| Husband | 77(55.8) | 7(2.5) | 84(20.3) |
| Alone | 1(0.7) | 11(4.0) | 12(2.9) |
| **Family size** |  |  |  |
| Three or less | 4(2.9) | 25(9.1) | 29(7.0) |
| Four-Six | 63(45.7) | 149(54.0) | 212(51.2) |
| Seven and above | 71(51.4) | 102(37.0) | 173(41.8) |
| **Father's education level** |  |  |  |
| None | 101(73.2) | 189(68.5) | 290(70.0) |
| Primary or above | 27(19.6) | 70(25.4) | 97(23.4) |
| Not known | 10(7.2) | 17(6.2) | 27(6.5) |
| **Mother's educational level** |  |  |  |
| None | 119(86.2) | 234(84.8) | 353(85.3) |
| Primary or above | 7(5.1) | 20(7.2) | 27(6.5) |
| Not known | 12(8.7) | 22(8.0) | 34(8.2) |

**Table 3:Socio-demographic and economic characteristics assessed as determinants of teenage pregnancy among female teenagers in Degua Tembien District, Tigray, Northern Ethiopia, 2015. (N=414) ........(Continued).**

| **Variables** |  | **Teenage** | **pregnancy** | **Total** |
| --- | --- | --- | --- | --- |
|  |  | **Cases** | **Controls** |  |
|  |  | **n (%)** | **n (%)** | **N (%)** |
| **Father's occupation** | |  |  |  |
| Farmer | | 136(98.6) | 258(93.5) | 394(95.2) |
| Other**#** | | 2(1.4) | 18(6.5) | 20(4.8) |
| **Mother's occupation** | |  |  |  |
| Farmer | | 133(96.4) | 239(86.6) | 372(89.9) |
| Other* | | 5(3.6) | 37(13.4) | 42(10.1) |
| **Monthly income of family in Ethiopian Birr(ETB)** | |  |  |  |
| Up to 500 ETB | | 21(15.2) | 10(3.6) | 31(7.5) |
| 500-1000 ETB | | 37(26.8) | 49(17.8) | 86(20.8) |
| 1000-2000 ETB | | 39(28.3) | 105(38.0) | 144(34.8) |
| 2001-3000 ETB | | 20(14.5) | 60(21.7) | 80(19.3) |
| > 3000 ETB | | 21(15.2) | 52(18.8) | 73(17.6) |
| **Watching TV/Listening to the radio at least once per week** | |  |  |  |
| Yes | | 54(39.1) | 145(52.5) | 199(48.1) |
| No | | 84(60.9) | 131(47.5) | 215(51.9) |
| **Time required to reach health facility** | |  |  |  |
| One hour or below | | 79(57.2) | 187(67.8) | 266(64.3) |
| Greater than one hour | | 59(42.8) | 89(32.2) | 148(35.7) |
| **Time required to reach school** | |  |  |  |
| One hour or below | | 108(78.6) | 197(71.4) | 305(73.7) |
| Greater than one hour | | 30(21.7) | 79(28.6) | 109(26.3) |

**#** Merchant, daily laborer, government employee.

***** Housewife, merchant, daily laborer, government employee.

**Table 4: Reproductive health characteristics assessed as determinants of teenage pregnancy among female teenagers in Degua Tembien District, Tigray, Northern Ethiopia, 2015. (N=414)**

| **Variables** | | **Teenage pregnancy** | |  |
| --- | --- | --- | --- | --- |
|  |  |  |  |  |
|  |  | **Cases** | **Controls** | **Total N(%)** |
|  |  | **n (%)** | **n (%)** |  |
| **Maternal history of teenage pregnancy** | |  |  |  |
| Yes | | 105(76.1) | 158(57.2) | 263(63.5) |
| No | | 33(23.9) | 118(42.8) | 151(36.5) |
| **Sister has history of teenage pregnancy** | |  |  |  |
| Yes | | 29(21.0) | 68(24.6) | 97(23.4) |
| No | | 109(79.0) | 208(75.4) | 317(76.6) |
| **Past history of contraception use** | |  |  |  |
| Yes | | 24(17.4) | 49(79) | 73(36.5) |
| No | | 114(82.4) | 13(21) | 127(63.5) |
| **Knowledge of occurrence of teenage pregnancy** | | |  |  |
| Good | | 65(47.1) | 174(63.0) | 239(57.7) |
| Poor | | 73(52.9) | 102(37.0) | 175(42.3) |
| **Communication with parents on sexual issues** | | |  |  |
| Yes | 51(37.0) | | 186(67.4) | 237(57.2) |
| No | 87(63.0) | | 90(32.6) | 177(42.8) |
| **Menarche** |  | |  |  |
| Yes | 137(99.3) | | 217(78.6) | 354(85.5) |
| No | 1(0.7) | | 59(21.4) | 60(14.5) |
| **Age at menarche** |  | |  |  |
| 10-13 years | 1(0.7) | | 14(6.5) | 15(4.2) |
| 14-16 years | 119(86.9) | | 187(86.2) | 306(86.4) |
| 17-19 years | 17(12.4) | | 16(7.3) | 33(9.3) |
| **Received information on menstruation** |  | |  |  |
| Yes | 123(89.1) | | 252(91.3) | 375(90.6) |
| No | 15(10.9) | | 24(8.7) | 39(9.4) |
| **Time received information on menstruation** | | |  |  |
| Before menarche | 87(70.7) | | 207(82.1) | 294(78.4) |
| After menarche | 36(29.3) | | 45(17.9) | 81(21.6) |
| **Age at sexual debut** |  | |  |  |
| 10-15 years | 3(5) | | 0(0) | 3(5) |
| 16-17 years | 34(56.7) | | 13(21.7) | 47(78.3) |
| 18-19 years | 2(3.3) | | 8(13.3) | 10(16.7) |
| **Sex education at school** |  | |  |  |
| Received | 22(18.5) | | 183(70.4) | 205(54.1) |
| Not received | 97(81.5) | | 77(29.6) | 174(45.9) |
| **Past pregnancy** |  | |  |  |
| Yes | 12(8.7) | | 0(0) | 12(2.9) |
| No | 126(91.3) | | 276(100) | 402(97.1) |

**Table 5: Reproductive health related characteristics assessed as determinants of teenage pregnancy among pregnant female teenagers in Degua Tembien district, Tigray, Northern Ethiopia, 2015. (n=138)**

| **Variables** | **Teenage pregnancy** | |  |
| --- | --- | --- | --- |
|  |  |  | **Total** |
|  | **Cases** | **Controls** | **N(%)** |
|  | **n (%)** | **n(%)** |  |
| **Past pregnancy** |  |  |  |
| Intended | 2(16.7) |  | 2(16.7) |
| Unintended | 10(83.3) | NA | 10(83.3) |
| **Age at first pregnancy** |  |  |  |
| 10-15 years | 1(0.7) |  | 1(0.7) |
| 16-17 years | 24(17.4) |  | 24(17.4) |
| 18-19 years | 113(81.9) | NA | 113(81.9) |
| **Current pregnancy** |  |  |  |
| Intended | 81(58.7) |  | 81(58.7) |
| Unintended | 57(41.3) | NA | 57(41.3) |
| **Initiation of ANC** |  |  |  |
| Yes | 129(93.5) |  | 129(93.5) |
| No | 9(6.5) | NA | 9(6.5) |
| **Time of ANC registration** |  |  |  |
| Before/at 16 weeks | 63(48.8) |  | 63(48.8) |
| After 16 weeks | 66(51.2) | NA | 66(51.2) |

NA: Not applicable

**Table 6: Pregnancy knowledge of female teenagers in Degua Tembien District, Tigray, Northern Ethiopia, 2015. (n=414)**

| **Variable** | **Teenage pregnancy** | | |
| --- | --- | --- | --- |
|  | **Cases** | **Controls** | **Total** |
|  | **n (%)** | **n (%)** | **N (%)** |
| **Knew the danger period for getting pregnant** | |  |  |
| Yes | 61(44.2) | 150(54.3) | 211(51.0) |
| No | 77(55.8) | 126(45.7) | 203(49.0) |
| **Knew at least two complications of teenage pregnancy** | |  |  |
| Yes | 46(33.3) | 133(48.2) | 179(43.2) |
| No | 92(66.7) | 143(51.8) | 235(56.8) |
| **Knew at least two prevention methods of teenage pregnancy** | |  |  |
| Yes | 9(6.5) | 28(10.1) | 37(8.9) |
| No | 129(93.5) | 248(89.9) | 377(91.1) |
| **Heard of modern contraception methods** |  |  |  |
| Yes | 126(91.3) | 229(83.0) | 355(85.7) |
| No | 12(8.7) | 47(17.0) | 59(14.3) |
| **Can list at least two types of contraception** |  |  |  |
| Yes | 122(88.4) | 223(80.8) | 345(83.3) |
| No | 16(11.6) | 53(19.2) | 69(16.7) |
| **Knew the double advantage of condom use** |  |  |  |
| Yes | 87(63.0) | 216(78.3) | 303(73.2) |
| No | 51(37.0) | 60(21.7) | 111(26.8) |
| **Knew at least two sites where contraception was available** | |  |  |
| Yes | 7(5.1) | 16(5.8) | 23(5.6) |
| No | 131(94.9) | 260(94.2) | 391(94.4) |
| **Overall knowledge status** |  |  |  |
| Good | 65(47.1) | 174(63.0) | 239(57.7) |
| Poor | 73(52.9) | 102(37.0) | 175(42.3) |

**Table 7: Logistic regression analysis of selected variables assessed as determinants of teenage pregnancy among female teenagers in Degua Tembien District, Tigray, Northern Ethiopia, 2015.(n=414)**

|  | **Teenage pregnancy** | |  |  | |  | |  | | | | | |  |  |  |
| --- | --- | --- | --- | --- | --- | --- | --- | --- | --- | --- | --- | --- | --- | --- | --- | --- |
| **Variables** | **Cases** | **Controls** | **COR(95%CI)** |  | | | |  | | | | | |  |  |  |
|  | **n (%)** | **n (%)** |  | **AOR(95%CI)** | |  | |  |  |  |  |  |  |  |  |  |
| **Age group of participant** |  |  |  |  | |  | |  | | | | | |  |  |  |
| 13-15 years | 1(0.7) | 32(11.6) | 0.33(0.04 -2.64) | 0.43(0.04-4.51) | |  | |  | | | | | |  |  |  |
| 16-17 years | 12(8.7) | 127(46.0) | 1 | 1 | |  | |  | | | | | |  |  |  |
| 18-19 years | 125(90.6) | 117(42.4) | 11.3 (5.94-21.52)* | **16.75(6.45-43.47)**** | |  | |  | | | | | |  |  |  |
| **Marital status** |  |  |  |  | |  | |  | | | | | |  |  |  |
| Single | 39(28.3) | 235(85.1) | 1 | 1 | |  | |  | | | | | |  |  |  |
| Married | 97(70.3) | 34(12.3) | 17.19(10.20-28.83)* | **15.91(7.43-34.04)**** | |  | |  | | | | | |  |  |  |
| Divorced | 2(1.4) | 7(2.5) | 1.72(0.35-8.59) | 1.30(0.16-10.73) | |  | |  | | | | | |  |  |  |
| **Father's occupation** |  |  |  |  | |  | |  | | | | | |  |  |  |
| Farmer | 136(98.6) | 258(93.5) | 4.46(1.02-19.60)* | 1.48(0.20-10.89) | |  | |  | | | | | |  |  |  |
| Other | 2(1.4) | 18(6.5) | 1 | 1 | |  | |  | | | | | |  |  |  |
| **Mother's occupation** |  |  |  |  | |  | |  | | | | | |  |  |  |
| House wife | 133(96.4) | 239(86.6) | 2.191(0.81-5.94) | 1.52(0.31-7.53) | |  | |  | | | | | |  |  |  |
| Other | 5(3.6) | 37(13.4) | 1 | 1 | |  | |  | | | | | |  |  |  |
| **Monthly income** |  |  |  |  | |  | |  | | | | | |  |  |  |
| Up to 500 ETB | 21(15.2) | 10(3.6) | 5.20(2.10-12.89)* | **23.96(4.89-117.29)**** | | | | |  | | |  | | | |  |
| 500-1000 ETB | 37(26.8) | 49(17.8) | 1.87(0.96-3.63) | **4.91(1.64-14.66)**** | | |  | | | |  | | | |  |  |
| 1001-2000 ETB | 39(28.3) | 105(38.0) | 0.92(0.49-1.72) | 2.52(0.90-7.06) | | |  | | | |  | | | |  |  |
| 2001-3000 ETB | 20(14.5) | 60(21.7) | 0.83(0.40-1.69) | 1.39(0.46-4.15) | | |  | | | |  | | | |  |  |
| > 3000 ETB | 21(15.2) | 52(18.8) | 1 | 1 | | |  | | | |  | | | |  |  |
| **Watching TV /Listening to radio at least weekly** |  |  |  |  | |  | |  | | | | | |  |  |  |
| Yes | 54(39.1) | 145(52.5) | 1 | 1 | |  | |  | | | | | |  |  |  |
| No | 84(60.9) | 131(47.5) | 1.72(1.14-2.61)* | 1.42(0.70-2.87) | |  | |  | | | | | |  |  |  |
| **Time required to reach health facility** |  |  |  |  | |  | |  | | | | | |  |  |  |
| One hour or below | 79(57.2) | 187(67.8) | 1 | 1 | |  | |  | | | | | |  |  |  |
| Greater than one hour | 59(42.8) | 89(32.2) | 1.57(1.03-2.39)* | 1.014(0.47-2.17) | |  | |  | | | | | |  |  |  |
| **Maternal history of teenage pregnancy** |  |  |  |  | |  | |  | | | | | |  |  |  |
| No | 33(23.9) | 118(42.8) | 1 | 1 | |  | |  | | | | | |  |  |  |
| Yes | 105(76.1) | 58(57.2) | 2.38(1.50-3.76)* | **4.14(1.84-9.33)**** | |  | |  | | | | | |  |  |  |
| **Knowledge of getting pregnant** |  |  |  |  | |  | |  | | | | | |  |  |  |
| Good | 65(47.1) | 174(63.0) | 1 | 1 | |  | |  | | | | | |  |  |  |
| Poor | 73(52.9) | 102(37.0) | 1.92(1.27-2.90)* | 1.44(0.68-3.04) | |  | |  | | | | | |  |  |  |
| **Communication with parents on RH issues** |  |  |  |  | |  | |  | | | | | |  |  |  |
| Yes | 51(37.0) | 186(67.4) | 1 | | 1 | | | | |  | | |  | | | |
| No | 87(63.0) | 90(32.6) | 3.53(2.30-5.41)* | | **6.52(3.12-13.64)**** | | | | |  | | |  | | | |

*Significant at p<0.05 ** Predictors at p < 0.001

**Table 8: Logistic regression analysis of selected variables assessed as determinants of teenage pregnancy among female teenagers in Degua Tembien District, Tigray, Northern Ethiopia, 2015. (n=414).....(Continued).**

| **Variables** | **Teenage pregnancy** | | **COR( 95%CI)** | **AOR(95%CI)** |
| --- | --- | --- | --- | --- |
|  | **Cases** | **Controls** |  |  |
|  | **n (%)** | **n (%)** |  |  |
| **Highest education level** |  |  |  |  |
| None- Grade 4 | 58(42.0) | 46(16.7) | 16.39(2.07-129.96)* | 20.24(0.80-512.07) |
| Grade 4-Grade 8 | 68(49.3) | 145(52.5) | 6.10(0.78-47.56) | 9.08(0.39-212.13) |
| Grade 9-Grade 10 | 11(8.0) | 72(26.1) | 1.99(0.24-16.72) | 2.59(0.10-64.74) |
| Grade 11 and above | 1(0.7) | 13(4.7) | 1 | 1 |
| **Family size** |  |  |  |  |
| Three or less | 4(2.9) | 25(9.1) | 1 | 1 |
| Four-Six | 63(45.7) | 149(54.0) | 2.64(0.8-7.91) | 1.05(0.26-4.20) |
| Seven and above | 71(51.4) | 102(37.0) | 4.35(1.45-13.05)* | 0.92(0.23-3.77) |

*Significant at p<0.05
